# Ultrafast charge transfer dynamics in 2D covalent organic frameworks/Re-complex hybrid photocatalyst

**DOI:** 10.1038/s41467-022-28409-2

**Published:** 2022-02-11

**Authors:** Qinying Pan, Mohamed Abdellah, Yuehan Cao, Weihua Lin, Yang Liu, Jie Meng, Quan Zhou, Qian Zhao, Xiaomei Yan, Zonglong Li, Hao Cui, Huili Cao, Wenting Fang, David Ackland Tanner, Mahmoud Abdel-Hafiez, Ying Zhou, Tonu Pullerits, Sophie E. Canton, Hong Xu, Kaibo Zheng

**Affiliations:** 1 Department of Chemistry, Technical University of Denmark, DK-2800 Kongens, Lyngby, Denmark; 2 Chemical Physics and NanoLund, Lund University, Box 124, 22100 Lund, Sweden; 3 Department of Chemistry, Qena Faculty of Science, South Valley University, 83523 Qena, Egypt; 4 State Key Laboratory of Oil and Gas Reservoir Geology and Exploitation, Southwest Petroleum University, Chengdu, 610500 China; 5 Department of Energy Conversion and Storage, Technical University of Denmark, DK-2800 Kongens, Lyngby, Denmark; 6 Institute of Nuclear and New Energy Technology, Tsinghua University, Beijing, 100084 China; 7 Department of Physics and Astronomy, Uppsala University, 75237 Uppsala, Sweden; 8 National University of Science and Technology “MISiS”, Moscow, 119049 Russia; 9 European XFEL, Holzkoppel 4, 22869 Schenefeld, Germany

**Keywords:** Photocatalysis, Chemical physics, Reaction kinetics and dynamics, Nanoscale materials

## Abstract

Rhenium(I)-carbonyl-diimine complexes have emerged as promising photocatalysts for carbon dioxide reduction with covalent organic frameworks recognized as perfect sensitizers and scaffold support. Such Re complexes/covalent organic frameworks hybrid catalysts have demonstrated high carbon dioxide reduction activities but with strong excitation energy-dependence. In this paper, we rationalize this behavior by the excitation energy-dependent pathways of internal photo-induced charge transfer studied via transient optical spectroscopies and time-dependent density-functional theory calculation. Under band-edge excitation, the excited electrons are quickly injected from covalent organic frameworks moiety into catalytic Rhenium^I^ center within picosecond but followed by fast backward geminate recombination. While under excitation with high-energy photon, the injected electrons are located at high-energy levels in Rhenium^I^ centers with longer lifetime. Besides those injected electrons to Rhenium^I^ center, there still remain some long-lived electrons in covalent organic frameworks moiety which is transferred back from Rhenium^I^. This facilitates the two-electron reaction of carbon dioxide conversion to carbon monoxide.

## Abstract (web summary)

Re complexes within covalent organic frameworks have emerged as promising photocatalysts for CO_2_ reduction. Here, authors identify a high-energy electron transfer pathway during CO_2_ reduction that results in longer-lived excited states than a low-energy electron transfer pathway.

## Introduction

The solar-driven photocatalytic conversion is regarded as one of the most promising approaches for carbon dioxide (CO_2_) transformation to tackle the rising issue of greenhouse gas emission^1–7^. Among the state-of-the-art photocatalysts for CO_2_ reduction, Rhenium(I)-carbonyl-diimine complexes have attracted considerable attention due to their high photocatalytic quantum yield and selectivity for CO_2_ reduction^8–12^. In general, one photon excitation in the molecule can usually trigger one-electron transfer highly endergonic for CO_2_ reduction^10^. The one-electron reduced (OER) Re complexes is still energetically favorable to donate the second electron. This is vital for CO_2_ reduction which is usually mediated by a two-electron transfer process^8–14^. However, the short excited-state lifetime and accessible annihilation of multi-excitons in the molecules remains an obstacle for such a process. In addition, the intrinsic metal-to-ligand charge-transfer (MLCT) absorption transition in Re complexes covers a limited spectral region (350–450 nm)^13,15^. Therefore Re complexes are usually paired with suitable photosensitizer to provide efficient and long-lived excited electrons for photocatalytic reaction^16–19^. Computational studies of CO_2_ reduction have also been widely reported for the Re-complexes^20–24^ and heterogeneous systems^25,26^. One emerging strategy is to immobilize such Re-complexes into porous scaffolds such as covalent organic frameworks (COFs) to construct heterogeneous molecular photocatalysts^27–30^. COFs are porous crystalline polymeric materials constructed by covalently bonded organic building blocks with highly ordered and periodic network structures^31,32^. The extended π-conjugation ensures broad light absorption and high charge conductivity^28^. Equally important, the porous structure with the large surface area provides numerous active sites for CO_2_ capture and catalytic reaction^33,34^. Recent studies reported high photocatalytic CO_2_ reduction activity on such Re-complex/COFs hybrid systems, which were rationalized by the efficient intramolecular charge transfer (ICT) from COFs units to Re-complexes^28,29^ However, the detailed charge-transfer dynamics have not been thoroughly understood. In fact, the charge-transfer pathways in Re-complex/COFs hybrids can be complicated. Taking the two-dimensional (2D) donor–acceptor (2D D-A) COFs as an example, the high degree of π-conjugation imparts a semiconducting behavior while the polaron formation reduces the exciton binding energy^35,36^. In addition, the early-time (i.e., sub-picosecond to picosecond) excited-state dynamics in COFs comprises multi-steps inter-unit charge transfer. Such process may compete or modulate the ICT, which should occur within a similar time scale^35^.

Another imperative topic worth investigating is the dynamics of the hot carriers excited by the high-energy photons above the bandgap of the COFs. In other words, these charge carriers are located at an energy level higher than the band edge of the samples. We can define such charge carriers as ‘hot’ electrons or ‘hot’ holes. In traditional semiconductors, the hot carriers quickly thermalize to the band edge. Harvesting the energy of the hot carriers without losing it as thermal energy is a crucial step to break the 33% Shockley–Queisser thermodynamic efficiency limit of a standard single-junction solar cell^37^. The same gain in energy conversion efficiency can be expected in photocatalytic reactions^37–41^. Considering the existence of inter-unit charge transfer during the excited-state relaxation process in 2D D-A COFs which can be governed by the molecular assembly, the hot-carrier collection (i.e., charge transfer of the hot carriers to the catalytic site and participants in the catalytic reduction or oxidation process before they cool down to the lowest excited states within the catalysts) should be feasible in Re-complex/COFs but requires experimental evidence.

Herein, taking a Re(CO)_5_Cl incorporated 2D COFs (TpBpy, constructed from triformylphloroglucinol (Tp) and 2,2′-bipyridine (Bpy)), named Re-TpBpy as an example, we demonstrate the excited-state dynamics and charge-transfer process in the hybrid catalyst. The time-dependent density-functional theory (TD-DFT) calculations display the available electronic transition after excitation of the hybrid catalyst. Computational studies of CO_2_ reduction were reported for the Re-complexes^20–24^ and heterogeneous systems^25,26^. The femtosecond transient visible (fs-TA) and time-resolved infrared (fs-TRIR) absorption spectroscopies provide complementary information for the charge-transfer dynamics. Photons with energy close to the bandgap of the COFs directly excite the electron from ground state to the excited states of Bpy followed by sub-picosecond electron injection to Re center. However, the injected electrons rapidly undergo geminated recombination with the residual holes in the COFs moiety within 13 ps. When the excitation is well above the band edge, the hot electrons and holes are initially generated evenly crosswise the entire COFs. The hot electrons would directly inject into the higher energy orbital of Re center within 2 ps and rebound to the Bpy within 24 ps. The hot holes slowly relax to the highest occupied molecular orbital (HOMO) level of COFs (340 ps). The prolonged excited electron lifetime in Re center and the higher energy levels, together with the additional long-lived free electrons in COFs moiety contribute as merits for a two-electron transfer mediated CO_2_ catalytic reaction. Our study rationalizes the excitation energy-dependent photocatalytic reaction mechanism in such Re-complex/COFs hybrid system, which can be beneficial for future material engineering towards optimal photocatalytic performance.

## Results

**Fig. 1:**
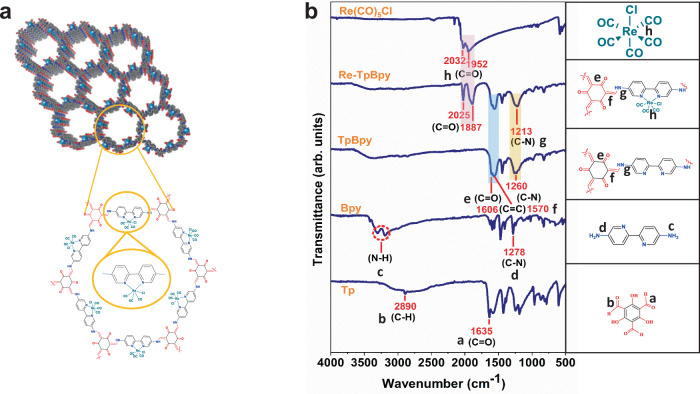
Chemical structures of the samples. (**a**) Schematic structure of the Re-TpBpy. (**b**) FT-IR spectra of TpBpy, Re-TpBpy and their starting materials with the right panel showing the molecular structure of the corresponding unit, where the bonds of a: C=O, b: C–H; c: N–H; d: C–N; e: C=O; f: C=C, and g: C–N are highlighted. Source data are provided as a Source data file.

Re-TpBpy is constructed by long-range ordered 2D sheets through the layer to layer stacking as shown in Fig. 1a. The characterization of TpBpy and Re-TpBpy was achieved by powder X-ray diffraction (PXRD), X-ray photoelectron spectroscopy (XPS), Fourier transform infrared (FT-IR), and nuclear magnetic resonance (NMR). PXRD patterns of both TpBpy and Re-TpBpy match well with the simulated AA stacking structure in the hexagonal space group (P6). After Re-complex incorporation, the crystalline structure of TpBpy remains unchanged. The XPS spectra confirmed the anchoring of Re-complex to the host TpBpy only through its bipyridinic units^30^ (for detailed XRD and XPS characterization, see Supplementary Figs. 2 and 3 in the supporting information). The ratio between N and Re was 5:1 according to the energy dispersive X-ray (EDX) characterization (Supplementary Fig. 8), which is slightly lower than the calculated full coordination of Re(I) as shown in Supplementary Fig. 1. The FT-IR spectrum (Fig. 1b) of TpBpy showed two new distinct peaks at 1606 (C=O) and 1570 (C=C) cm^−1^, which are the typical stretching frequency of the keto-form. Meanwhile, the concomitant absence of N–H stretching vibration (around 3300 cm^−1^ for Bpy) and the C=O stretching vibration (1635 cm^−1^) confirmed the complete transformation of starting materials^42^. On the other hand, the FT-IR spectrum of Re-TpBpy shows that the chemical functionalities present in the pristine TpBpy were preserved while two additional peaks arise at 2025 and 1887 cm^−1^ attributed to the C=O stretching vibration in the Re(CO)_3_Cl moiety. Furthermore, the C≡O stretching bonds (2025 and 1887 cm^−1^) and the C–N peak (1213 cm^−1^) of Re-TpBpy are slightly red-shifted compared with Re(CO)_5_Cl (2032 and 1952 cm^−1^) and TpBpy (1260 cm^−1^), indicating the coordination of Re(CO)_3_Cl to the bipyridinic N atoms in the TpBpy^30,43^, which can also be provided by solid ^13^C NMR (Supplementary Fig. 3).

**Fig. 2:**
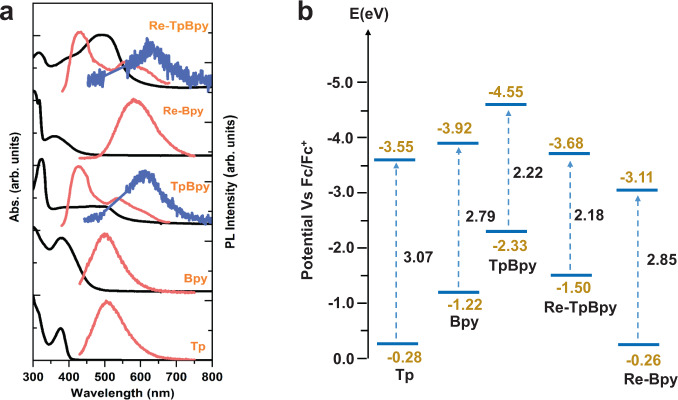
Steady-state spectroscopy characterization and energy band alignment. (**a**) Normalized UV−vis absorption (black) and steady-state photoluminescence spectra excited at 400 nm (red) and 530 nm (blue) of TpBpy, Re-TpBpy and their starting materials dispersed in Nafion (5% w/w in water and 1-propanol). (**b**) Band alignment established from both absorption spectroscopy and cyclic voltammetry. Source data are provided as a Source data file.

Steady-state absorption and photoluminescence (PL) spectra first clarified the ground state features of the Re-TpBpy hybrid catalyst and its individual units (Fig. 2a). The absorption spectra of the pure Tp and Bpy both showed a single distinct absorption band of Tp (350–400 nm) and S1 of Bpy (380 nm). The absorption spectrum of Re-Bpy is identical to Bpy except for a subtle blue shift (maxima absorption at 360 nm) owing to the MLCT [d(Re)-π*(bpy)]^44,45^. On the contrary, the absorption spectrum of TpBpy exhibits dual absorption bands with a narrow bipyridine n–π* transition band (324 nm) as well as a broad band at 508 nm for delocalized π electrons^46^. The similar absorption spectra between Re-TpBpy and TpBpy suggest the high ligand stability of TpBpy (i.e., chromophore function) after functionalization^30^. The slight blue shift of the absorption spectrum for Re-TpBpy due to the MLCT[d(Re)-π*(bpy)], which corresponds to the charge-transfer transition that has also been observed in the Re-bipyridine complexes^3,4^. We can further calculate the optical band gaps (*E*_g_^opt^) of Tp, Bpy, Re-Bpy, TpBpy and Re-TpBpy from the steady-state absorption (Fig. 2a) by the formula of *E*_g_^opt^ = 1240/*λ*_edge_ to be 3.07, 2.70, 2.85, 2.22, and 2.18 eV, respectively. The Cyclic voltammetry (CV) measurements were employed to investigate the electrochemical properties of these samples, and the voltammograms are shown in Supplementary Fig. 5. The HOMO and lowest unoccupied molecular orbital (LUMO) energy levels were calculated by combining both absorption spectroscopy and CV. The LUMO energy levels were calculated from the first reduction onset potential using as reported equation *E*_LUMO_ = − (*E*_red/onset_ + 4.4) eV and the HOMO energy levels were determined by the *E*_g_^opt^ plus LUMO energy levels^47–50^. Figure 2b summarized the band energy alignment of the samples and Supplementary Table 1 summarized the optical and electrochemical properties of samples.

Figure 2a also shows the emission spectra of all samples excited at the band edge (530 nm, blue curve) and well above the band edge (400 nm, red curve). Tp and Bpy share the same emission band at 500 nm upon the 400 nm excitation with moderate Stokes shift. In contrast, the emission spectrum of the Re-Bpy (λem = 585 nm) is broad with a large Stokes shift (Δλ = 225 nm), which can be attributed to the existence of a triplet metal-to-ligand charge-transfer (^3^MLCT) states^51^. When excited at 530 nm, the emission spectra of TpBpy and Re-TpBpy are identical with emission bands at 620 nm. This indicates similar emissive states from delocalized π electrons in the two samples. When excited at higher energy (400 nm), the emission spectra of TpBpy and Re-TpBpy exhibit dual emission bands (i.e., 427 nm and 531 nm for TpBy, 431 nm and 562 nm for Re-TpBpy). The origins of such multi-emission bands in COFs can be complicated where one hypnosis is the radiative recombination in the single units of the COFs^52^. We can obtain the same conclusion in the following analysis of the excited states. In summary, the optical transitions of TpBpy and Re-TpBpy are distinct from the ones of their original building block units.

**Fig. 3:**
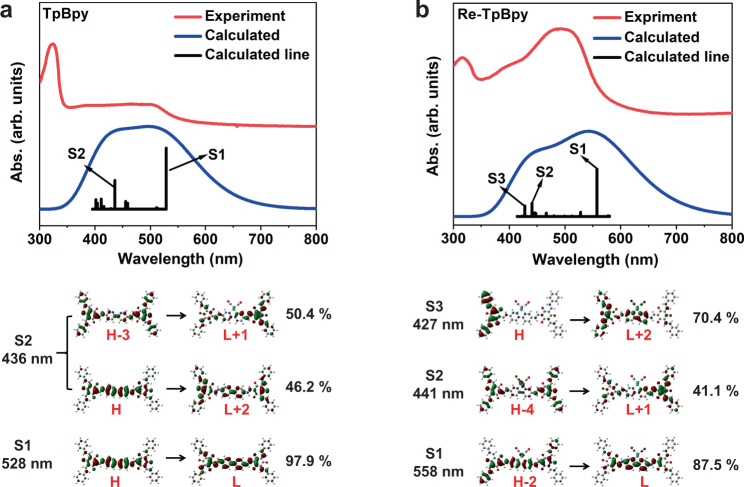
TD-DFT calculation of the samples. UV−vis absorption spectra of (**a**) TpBpy and (**b**) Re-TpBpy compared with TD-DFT calculated fragment. Source data are provided as a Source data file.

In order to obtain insight into the excited-state structure of the compounds, we used a triformylphloroglucinol (Tp) terminated bipyridine (Bpy) molecular fragment to represent the COFs structure (Supplementary Fig. 12). The TD-DFT at the M06-L^20,53–55^/def 2-TZVP^56,57^ level of theoryhas been employed to calculate the electronic structure and model the electronic transitions. Figure 3 exhibits the calculated electronic excitation spectra (blue curves) of the TpBpy and Re-TpBpy, which can resemble the experimental absorption spectra (red curves). The calculated spectrum of TpBpy mainly consists of two electronic excitation bands at 528 nm (S_1_) and 436 nm (S_2_), where high-energy band S_2_ is equally contributed by the electronic transition (HOMO-3 → LUMO + 1) and (HOMO → LUMO + 2). The low-energy band S_1_ is dominated by the electronic transition from HOMO to LUMO level as illustrated in Fig. 3c. The low-energy optical transition only occurs at the Bpy moiety whereas the high-energy transition involves the electron population at both Tp and Bpy in the COFs moieties. The modeled spectrum of Re-TpBpy (Fig. 3b) also shows two pronounced electronic excitation bands where the high-energy band is contributed by two electronic transition S_3_ (HOMO → LUMO + 2, 427 nm) and S_2_ (HOMO-4 → LUMO + 1, 441 nm). The low-energy band consists of one electronic transition S_1_ (HOMO-2 → LUMO + 1, 558 nm). Compared with TpBpy, the low-energy electronic transition in Re-TpBpy involves the excitation of the electron from the orbital in both Tp and Bpy moieties. The detailed calculated orbitals for both samples have been summarized in the supporting information.

**Fig. 4:**
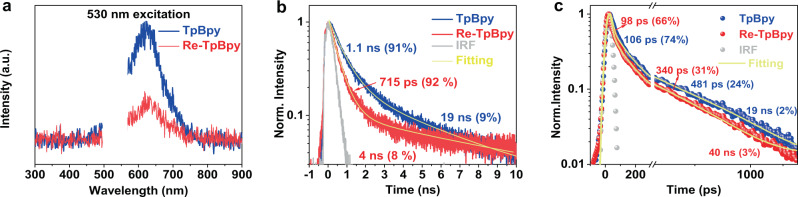
Steady-state and time-resolved PL spectroscopy measurement. (**a**) Steady-state PL emission spectra of TpBpy and Re-TpBpy normalized according to the absorbance at the excitation wavelength. (**b**) PL decays measured in TCSPC of the TpBpy and Re-TpBpy. Excitation wavelength = 438 nm. (**c**) PL decays of the TpBpy and Re-TpBpy measured with streak camera excited at 400 nm. Source data are provided as a Source data file.

In the next step, we studied the PL dynamics of the samples. The steady-state PL spectra of TpBpy and Re-TpBpy are identical in terms of emission energy and spectral shapes (Fig. 4a). However, the relative PL quantum yield (extracted from absorption calibrated PL intensities) of Re-TpBpy is much lower. This should be attributed to the PL quenching by the integration of the Re-complex. The shorter PL lifetime of Re-TpBpy measured from time-correlated single-photon counting (TCSPC) verifies the additional non-radiative process (Fig. 4b). The exponential fitting can resolve two components with lifetimes of 1.1 ns (91%), 19 ns (9%) for TpBpy, and 716 ps (92%), 40 ns (8%) for Re-TpBpy. However, the lifetime of the fast components (i.e., 1.1 ns for TpBpy and 716 ps for Re-TpBpy) are limited by the response function in the TCSPC measurement. Therefore streak camera technique was employed to explore the ultrafast process. A similar faster PL decay of Re-TpBpy than TpBpy can be observed in Fig. 4c. The PL decays can be fitted by tri-exponential functions. The two fast components can then be fitted as 106 ps (74%), 481 ps (24%) for TpBpy, and 98 ps (66%), 340 ps (31%) for Re-TpBpy.

**Fig. 5:**
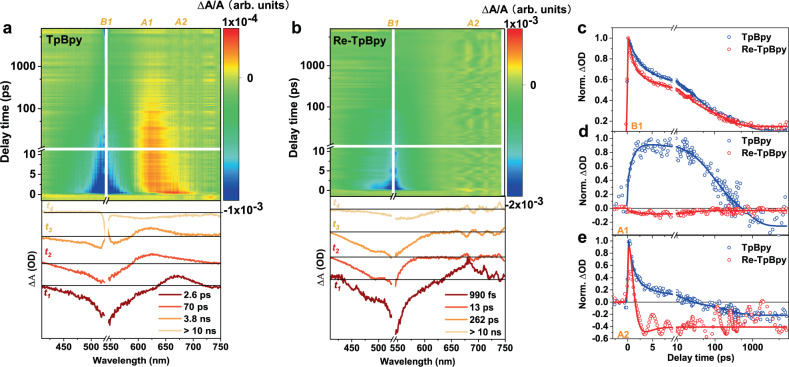
Transient absorption (TA) study at band-edge excitation. TA spectra under 530 nm excitation at the fluence of 2 × 10^13^ ph/cm^2^ and the respective SVD fitting results of TpBpy (**a**), and Re-TpBpy (**b**). TA kinetics at some characteristic wavelength of B1 at 535 nm (**c**), A1 at 625 nm (**d**), and A2 at 675 nm (**e**). All the samples were measured in Nafion (5% w/w in water and 1-propanol) solution. Source data are provided as a Source data file.

The excited-state dynamics of the samples were explored by transient absorption (TA) spectroscopy. We first excited the samples close to their band edge at 530 nm. In this case, all the excited species should populate the lowest excited states instantly. The TA spectra of TpBpy exhibit one broad negative band (B1) from 450 to 595 nm attributed to the band-edge ground state bleach (GSB) together with two positive excited-state absorption bands (ESA, A1 and A2) from 600 to 700 nm (Fig. 5a). According to the above DFT calculation in Fig. 3a, 530 nm excitation will only trigger the transition to the lowest excited state (i.e., HOMO to LUMO) in TpBpy. Hence, A1 and A2 here should not be attributed to different levels of the excited state. One possible explanation is the excited-state transform from a normal exciton state (A2) to a polaron state (A1) where the excitons are self-trapped within the local structure of the COFs^35^. Such polaron formation also complies with the significant stokes shift from the PL spectra, as shown in Fig. 2a.

For Re-TpBpy (Fig. 5b), only one B1 can be observed with the absence of long-lived ESA. This already indicates the charge transfer from the excited states. A more quantitative analysis was then implemented using singular value decomposition (SVD) fitting (Fig. 5a, b, lower panel). The TA dynamics of TpBpy can be decomposed into four decay-associated components (t_1_–t_4_). The first three components (t_1_–t_3_) shared the same negative GSB signal with the identical position (B1), denoting the population of the lowest excited state. The difference between the first component (t_1_ = 2 ps) with the second and third components (t_2_ = 70 ps and t_3_ = 4 ns) appears as the blue-shifted ESA band from A2 to A1 by about 50 nm. TA kinetics at the A1 (Fig. 5d) and A2 (Fig. 5e) reveal the concurrent rising of A1 and decay of A2. This indicates the transformation of the lowest excited state (e.g., polaron formation) within 2 ps corresponding to the transition of ESA from A2 to A1 in TA spectra. Components 2 and 3 exhibit the same spectral feature corresponding to the depopulation dynamics of the same lowest excited state. The slowest component 4 featured as broad negative band with a lifetime exceeding the TA time window. This can also be visualized in the TA kinetics in Fig. 5c–e. After functionalization by the Re-complex, the TA dynamics of Re-TpBpy can also be decomposed into four components (t_1_–t_4_) (Fig. 5b) with lifetime of t_1_ = 990 fs, t_2_ = 13 ps, t_3_ = 262 ps accompany with one ultra-long component. Component t_1_–t_3_ of Re-TpBpy resembles the GSB feature as TpBpy but with shorter lifetimes as evidenced by the comparison of B1 kinetics in Fig. 5c. Most importantly, ESA bands are completely absent in components t_2_ and t_3_. This suggests the ultrafast charge transfer from the lowest excited state. In addition, the longest component t_4_ also exhibits narrower GSB at the band-edge position compared with GSB in component t_4_ of TpBpy.

**Fig. 6:**
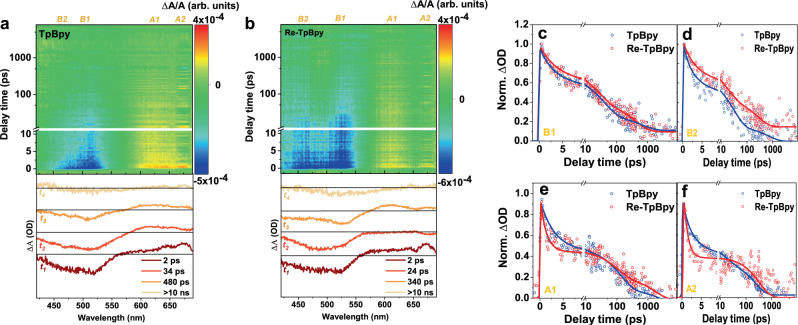
TA study at high-energy excitation. TA spectra under 400 nm excitation at the fluence of 2 × 10^13^ ph/cm^2^ and the respective SVD fittings of TpBpy (**a**), Re-TpBpy (**b**). TA kinetics of two samples at various emission wavelengths representing B1 (**c**), B2 (**d**), A1 (**e**), A2 (**f**). All spectra are recorded in Nafion (5% w/w in water and 1-propanol). Source data are provided as a Source data file.

In order to monitor the dynamics of hot carriers, high-energy excitation has also been employed in both samples. Compared with TA spectra excited at 530 nm, here the TA spectra of both TpBpy and Re-TpBpy (Fig. 6a, b) exhibit one additional negative band (B2) around 450 nm with the slight red-shifted B1 to 515 nm. Since B2 appears in both TpBpy and Re-TpBpy, the additional bleach band should be attributed to the population of high-energy/hot levels in the COFs unit. SVD fitting indicates that the dynamics of TpBpy can be described by four main components. The fastest component t_1_ (2 ps) consists of B1, B2, and A1. Component t_2_ (34 ps) features the same B1 and B2 bands but the ESA is blue-shifted to A2. The component t_3_ (480 ps) lifetime shares almost the same spectral features of component 2 except for the absence of B2. A similar lifetime (481 ps) can also be extracted in the PL decay of TpBpy (Fig. 4c), manifesting the radiative recombination of the band-edge charge carriers. The component t_4_ only contains B1 but the contribution is negligible. The above SVD analysis indicates the long-lived B1 versus short-lived B2 as also evidenced by the extracted TA kinetics in Fig. 6c and d (blue curve).

On the other hand, both A1 and A2 appear instantaneously which is different from the one observed for 530 nm excitation. TA spectra of Re-TpBpy can also be fitted with four main components (t_1_ = 2 ps, t_2_ = 24 ps, t_3_ = 340 ps and one ultra-long component). The features of component t_1_ resemble those of TpBpy with a similar lifetime of 2 ps. Compared with 530 nm excitation, B2 with 400 nm excitation are long-lived in both t_1_–t_3_ up to 340 ps. The prolonged B2 band in Re-TpBpy suggests that the long-lived high-energy level population in contrast to TpBpy as further illustrated by the TA kinetics in Fig. 6d. Furthermore, A1 disappears in component t_2_ and reoccurs in t_3_. This is consistent with the different A1 kinetics (Fig. 6e, red curve) from the B1 and B2 kinetics (Fig. 6c, d, red curve) especially at the timescale between 5 and 20 ps. The absence of A1 in component t_2_ can be induced by two possible scenarios: (1) there exist two pools of Re-TpBpy where electron transfer from TpBpy to Re^I^ occurs in one pool and absent in the other. (2) the charge transfer of hot electrons from the COFs to Re^I^ centers is followed by the backtransfer to the LUMO level. In the following, we will demonstrate the latter is more likely evidenced by time-resolved IR spectroscopy results, which probes the transient population of electrons at Re^I^ centers. The lifetime t_3_ (340 ps) can be obtained from the time-resolved photoluminescence (TRPL) decay in Fig. 4c, manifesting radiative recombination with hot carriers, which accounts for the high-energy emission band in the steady-state PL spectrum (Fig. 2a). Identical to TpBpy, component 4 of Re-TpBpy comprises only B1 with negligible amplitude. In short, the additional B2 band and the wider ESA band when excited at 400 nm in components t_1_, t_3_ reflect the long-live hot excited level population. On the other hand, the absence of A1 in component t_2_ confirms the charge transfer of hot electrons to Re^I^ centers within 2 ps.

**Fig. 7:**
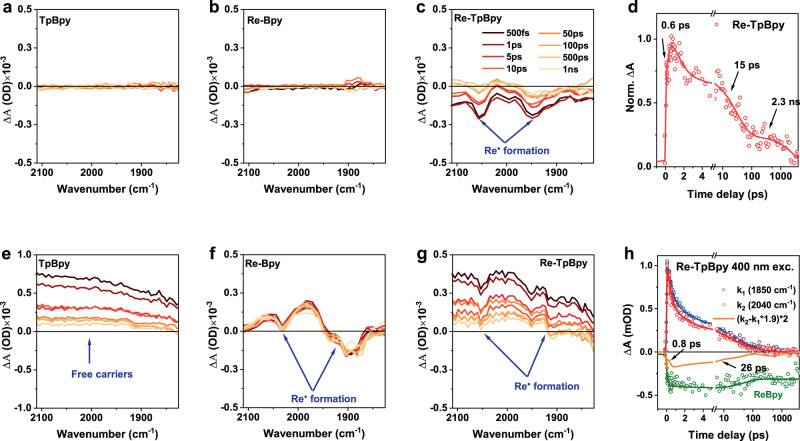
TRIR study of the samples. TRIR spectra of TpBpy, Re-Bpy, and Re-TpBpy excited at 530 nm (**a**–**c**) and 400 nm (**e**–**g**). TRIR kinetics at 2040 cm^−1^ of Re-TpBpy excited at 530 nm. (**h**) TRIR kinetics at 2040 cm^−1^ (red), 1850 cm^−1^ (blue) and their differential curve (orange) of Re-TpBpy excited at 400 nm. The kinetics at 2040 cm^−1^ of Re-Bpy excited at 400 nm is also presented (green). All spectra are recorded in Nafion (5% w/w in water and 1-propanol). Source data are provided as a Source data file.

In order to further characterize the excited-state dynamics at the two excitation wavelengths, we measured the time-resolved infrared spectroscopy (TRIR) spectra of the samples. TRIR can probe photo-induced electronic transitions at low energy such as molecular vibrations or intraband free carriers^58–62^. No TRIR signal can be observed in neither TpBpy nor Re-Bpy when excited at 530 nm (Fig. 7a, B). The TRIR spectrum of Re-TpBpy (Fig. 7c) exhibits pronounced ground state bleach at 1950 and 2040 cm^−1^ together with excited-state absorption band at around 2000 cm^−1^, similar to the spectral feature of pure Re-Bpy (Fig. 7f) excited at 400 nm. Such spectral features are the fingerprint of excited [ReI(bpy)(CO)3]*^63^. The TRIR kinetics in Fig. 7d suggests that the Re^I^ radical is formed within 0.6 ps (rising time of the kinetics) together with 2 decay lifetimes (15 ps and 2.3 ns). Such formation time of the Re^I^ radical is consistent with t_1_ in TA components (0.99 ps) (Fig. 5b), confirming the sub-picosecond electron transfer from TpBpy to the Re^I^ center after excitation. The 15 ps decay lifetime is identical to the component t_3_ in TA (Fig. 5b). When excited at 400 nm, the TRIR spectra of both TpBpy and Re-TpBpy (Fig. 7e, g) are dominated by the featureless positive absorption, which is widely accepted as the sign of free carrier generation in semiconductor materials^58,64,65^. This means that the hot excited states reflected by the B2 and the broad A1 band in TA should all be populated by free carriers when excited at 400 nm. Moreover, the TRIR spectrum in Re-TpBpy features additional differential dips of the Re^I^ radical, indicating the COFs-Re electron transfer occurs. We can decompose the dynamics of Re^I^ radical (orange curve, Fig. 7h) by subtracting the TA kinetics at such mixed region (2040 cm^−1^, red curve, Fig. 7h) by the kinetics at the region only showing positive absorption (1850 cm^−1^, blue curve, Fig. 7h). Here the intensity of TA kinetics at 1850 cm^−1^ is scaled up by the amplitude ratio between 1850 and 2040 cm^−1^ as extracted in Fig. 7g (*A*_2040 cm−1_/*A*_1850 cm−1_ = 1.9) with only free carrier contribution in COFs. The deferential kinetics (i.e., the orange curve in Fig. 7h) shows a 0.8 ps building up time followed by a 26 ps decay, which is consistent with the above argumentation that the hot electrons are injected to Re^I^ center within the picosecond and rebound to the S_1_ level of TpBpy in 26 ps. We also notice that such kinetics is different from the depopulation of photo-excited pure [Re^I^(bpy)(CO)3]* (green curve, Fig. 7h). This means the backtransfer or geminate recombination of injected electrons in Re^I^ center is faster than the electron-hole recombination in the Re^I^(bpy) moiety.

## Discussion

**Fig. 8:**
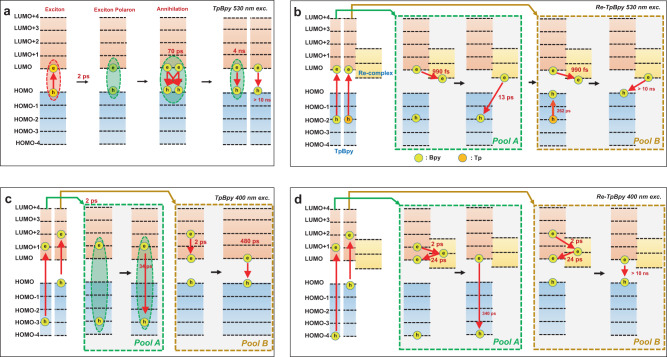
Summary on the excited-state dynamics. Schematic diagram of the pathway and lifetime excited-state dynamics of TpBpy and Re-TpBpy under (**a**) (**b**) 530 nm, and (**c**), (**d**) 400 nm excitation.

The TD-DFT calculations indicate the 530 nm excitation can only generate an electronic transition from HOMO to LUMO (S_1_) in TpBpy, which merely locates at Bpy moiety (Fig. 3a). The TRIR results further suggest that the dominant excited species are excitons. Such bounded exciton formation is reasonable as the photo-generated electrons and holes are close in space in Bpy. The ESA transition from A2 to A1 in TA spectra in Fig. 5a should represent the formation of exciton polarons from initially generated excitons. The following up excited-state depopulation at hundreds of ps can be observed in both the TA and TRPL results. We confirmed that the lifetime of such a process is highly dependent upon the excitation intensity (for details, see Supplementary Fig. 8b). This indicates the high order recombination of excited singlet excitons, which is often observed in conjugated polymers and semiconductor nanostructrues^66–69^. Previous research reports such ultrafast singlet-singlet exciton annihilation in COFs materials followed by the formation of ultra-long-lived specially separated charges^36^. However, the absence of free carriers over the whole time window suggests that the residual excited-state species are still excitons instead of free carriers in our COFs. However, the lifetime of such excitons varies according to the relative spatial location. The short lifetime (4 ns) should refer to the excitonic recombination within the same COFs sheet, while the ultra-long-lived exciton may contain the electrons and holes at a different sheet of COFs^36,70^. Figure 8a summarizes the observed excited-state dynamics.

In Re-TpBpy, the electronic transition from the HOMO-2 to the LUMO (Fig. 3b) corresponds to a partial charge-transfer state as the HOMO-2 locates at the orbitals over both Tp and Bpy moiety according to the calculation. The sub-picosecond depopulation of excited states in the TA spectra combined with rising of transient Re^I^* radical formation spectra in TRIR can be unambiguously assigned to the electron transfer from the LUMO located at Bpy to the Re^I^ center within 0.8–0.9 ps as shown in Fig. 8b. However, as holes can reside both at Tp and Bpy, we can expect different depopulation pathways of those holes after electron injection corresponding to two-lifetime components in TA (t_2_ and t_3_) (Fig. 5b). Holes at Bpy should undergo fast germinate recombination with electrons at Re^I^ center due to the short distance between electrons and holes (13 ps), which is consistent with the same decay component (15 ps) in TRIR (Fig. 7d) demonstrating the concurrent depopulation of electrons. On the other hand, those holes at Tp should be implausible to recombine with remote electrons at Re^I^ directly, instead, they will cool down to the HOMO level with relatively long time (262 ps) which is still strongly localized in Tp (for detailed electronic structure see Supplementary Table 3). Such slow cooling time may be due to the necessary inter-unit (e.g., Tp to Bpy, or Bpy to Tp) charge transfer in the cooling pathway. Therefore recombination between those holes and electrons in Re^I^ center will be inefficient, corresponding to the long component in both TA (Fig. 5b) and TRIR (Fig. 7d). The above analysis suggests the low-energy excitation in Re-TpBpy is very close to the Re-COF interface, thus facilitating the electron injection from the COFs to the Re^I^ center. However, geminate recombination is also efficient due to the close spacing between the injected electron and residual holes in COFs moiety, as summarized in Fig. 8b. In addition, according to the TD-DFT calculation, to maintain the symmetry and stability of the framework, the Re(I) should be alternatively pointed out to the different pore voids in the COFs structure, which results in the shortest Re-Re distance 2.18 nm as illustrated in Supplementary Fig. 13. Such distance is much longer than the average annihilation radius of COFs materials (1 nm)^71^, which means the inter-metal center annihilation is less likely to occur here.

TD-DFT calculation demonstrates at high-energy photon excitation condition, TpBpy will have a transition from HOMO → LUMO + 2 and HOMO-3→LUMO + 1 (Fig. 3a), while Re-TpBpy will exhibit an electronic transition from HOMO → LUMO + 2 and HOMO-4→LUMO + 1 (Fig. 3b). Compared with low-energy excitation, the excited states are distributed more evenly through the Tp and Bpy. In addition, the TRIR results suggest that the initially excited species include free carriers resided at those high-energy levels. The initial fastest component t_1_ in TA measurement (Fig. 6a) should be related to the partial polaron formation where some of the generated species also remain as free carriers as evidenced by the remaining A2 after 2 ps and the free carries absorption in the TRIR spectra. The dual emission band in steady-state PL of Fig. 2a supports that the excited-state depopulation should involve two parallel processes with radiative recombination from the higher level and lowest excited state. In TpBpy, the population of hot electrons at the LUMO + 2 and the LUMO + 1 as well as hot holes at the HOMO-3 contributed to the B2 in Fig. 6a while hole population at HOMO leads to the B1 consistent with the band-edge GSB at 530 nm excitation. t_2_ component in TA (Fig. 6a) features concurrent B1 and B2 should then be attributed to the radiative recombination between the electron at LUMO + 1 and hole at HOMO-3 leading to the high-energy PL emission. t_3_ component, on the other hand, should be attributed to the recombination between electrons and holes relaxed to the HOMO and LUMO level. Figure 8c summarizes the corresponding excited-state dynamics.

As summarized in Fig. 8d, at Re-TpBpy, the excited electron at LUMO + 2 and LUMO + 1 would be injected to Re^I^ center within 2 ps and then recombines to HOMO at Bpy within 24 ps resolved by the complementary dynamics observed in the TA and TRIR measurements, as shown in Fig. 8d. This can be due to either the hot-electron injection through the higher excited states in Re^I^ center or to the formation of a charge-transfer state (CTS) due to the Coulombic attraction between injection electron in Re^I^ and residual hole in COFs followed by the dissociation of such CTS. Both pathways are well-observed in other molecular or semiconductor systems^72,73^. Such a 24 ps lifetime for such intermediate excited state can be a merit for hot-carrier harvesting. After hot-carrier cooling, the depopulation of the excited states also depends on the spatial location of the charge carriers. If the hole locates at Bpy orbitals (HOMO-4 level at S_2_ in Fig. 3b), the hot-carrier emission will occur similarly to the case of TpBpy with lifetime of 340 ps corresponding to the t_3_ component observed in TA (Fig. 6b), since the hot hole cooling to the HOMO in Re-TpBpy is hindered by the additional Bpy-to-Tp charge transfer. If hole locates at Tp orbitals in HOMO (S_3_ in Fig. 3b), it will recombine with the relaxed electron at LUMO both radiatively and nonradiatively but with a longer lifetime as observed in both the TA and TRPL spectra.

**Fig. 9:**
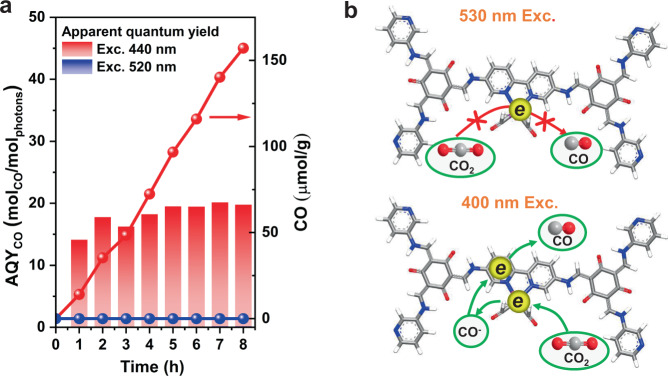
Photocatalytic performance. Photocatalytic evolutions of CO by Re-TpBpy under 520 and 440 nm excitation (**a**) and schematic diagram to rationalize the catalytic performance under two excitation conditions (**b**). Source data are provided as a Source data file.

The above analysis on the excited-state dynamics of TpBpy and Re-TpBpy suggests that anchoring Re-complex into COFs structure does facilitate the charge separation for the photocatalytic reduction process. However, when high excitation photon energy is used, efficient hot-electron injection would occur from TpBpy to higher energy orbital of Re-complex with the electron lifetime at Re^I^ center almost doubled compared to the lifetime with 530 nm excitation. In contrast to the conventional semiconductor where the photo-generated hot electrons will quickly dissipate energy and relax to the lowest excited state, the unique electronic structure of the Re-TpBpy catalyst is expected to boost the photocatalytic performance from hot carriers. The following CO_2_ photocatalytic reduction experiment confirmed our assumption. The evolution of CO by our Re-COFs catalyst exhibits a much higher yield when 440 nm excitation is used compared with band-edge excitation at 520 nm (Fig. 9a).

In order to verify the production of CO from the photocatalytic reaction of Re-COFs, the adsorbents from the Re-TpBpy surface are first measured by the mass spectra. As presented in Supplementary Fig. 14, after Re-TpBpy sample is treated at 120 °C under the Ar atmosphere, there are only signals indexed to H_2_O (*m/z* = 18) and CO_2_ (*m/z* = 44) detected in mass spectra. Therefore, it is suggested that there are only H_2_O and CO_2_ adsorption on the Re-TpBpy surface. Then, the suggested photochemical experiment in the absence of CO_2_ was carried out (Supplementary Fig. 15). It is found that the CO generation rate is 1.9 μmol g^−1^ h^−1^, which is much lower than that with the existence of CO_2_ (19.6 μmol g^−1^ h^−1^). The recycle photocatalytic CO_2_ reduction experiment in Supplementary Fig. 16 shows that Re-TpBpy sample reveals a similar CO generation rate across three runs. The above results identify that the obtained CO originated from the CO_2_ conversion and not from the desorption of adsorbents from the surface. We can quantify the catalytic performance by calibrating the CO yield with the absorbed light, which results in the apparent quantum yields (AQY) of the CO production as presented in Fig. 9a. The general AQY of our Re-COF catalysts is around 10 to 15% under high-energy excitation, while under band-edge excitation, the AQY is almost negligible.

In addition, we conducted the concentration-dependent PL quenching experiment. We found that the quenching time at the TEOA concentration provided in the catalytic measurement (0.5 M) for both 530 and 400 nm excitation are 3.5 and 0.1 ns. Both values are shorter than the longest component of the excited-state lifetime of pure Re-COF without TEOA from TA measurement, which is longer than 10 ns. This means that the hole scavenging process should still be feasible and efficient in the catalytic system before the Re-COF comes back to the ground state. The slower hole injection from 530 nm excitation compared with 400 nm excitation could be due to the different electronics structures for hole orbitals, which can probably also be one reason for the efficient catalytic performance under 400 nm excitation (Supplementary Fig. 17). The huge difference of CO production by Re-TpBpy under different wavelength irradiation should be attributed to (1) injected electrons are located at high-energy levels in Re^I^ centers with longer lifetime, which are favorable for the electron transfer process for the CO_2_ reduction, and (2) when excited with high-energy besides the injected electrons to Re^I^ center, there still remain long-lived electrons in COFs moiety which is transferred back from Re^I^. This makes the two-electron reaction of CO_2_ conversion to CO to work as illustrated in Fig. 9b.

In conclusion, we investigated the excited-state dynamics with focus on the ultrafast charge transfer in Re^I^(bpy)(CO)_3_/TpBpy hybrid photocatalyst by complementary time-resolved laser spectroscopies and numerical methods. We first determine the electronic transition of the hybrid structure using time-dependent DFT calculations to model the optical absorption. We found that the absorption spectrum of Re-TpBpy mainly consists of two bands with the low-energy bands contributed by the transition from ground state to excited state barely in Bpy moiety and the high-energy bands features the unoccupied orbital is contributed evenly by the whole COFs moiety. Combining the observations of the excited dynamics resolved both in TA and TRIR, entirely different inter-unit charge-transfer pathways in Re-TpBpy can be identified. Under band-edge excitation, the electrons excited at the HOMO level would quickly injected into Re^I^(bpy)(CO)_3_Cl within ps timescale and recombine within ns with the holes residing in Bpy close to Re center at about 13 ps. Under excitation with high-energy photon, the photo-generated hot electron is first injected into the highly excited level of Re^I^(bpy)(CO)_3_ within 1–2 ps and recombine to the HOMO in COFs within 24 ps. In addition, there remain long-lived free carriers in the COFs moiety. This can rationalize the good photocatalytic CO_2_ reduction performance of the obtained catalysts.

## Methods

### Materials and methods

5,5′-diamino-2,2′-bipyridine (95%, Yuhao Chemical), pentacarbonylchlororhenium (98%, Sigma-Aldrich), 2,2′-bipyridine (Bpy) (≥99%, Sigma-Aldrich), triformylphloroglucinol (Tp) (95%, Yuhao Chemical), mesitylene (98%, Sigma-Aldrich), 1,4-dioxane (anhydrous, 99.8%, Sigma-Aldrich), glacial acetic acid (ACS reagent, Aldrich), Nafion (10 wt% in H_2_O), 1-propanol (for HPLC, ≥99.9%, Sigma-Aldrich), toluene (for HPLC, VWR Chemicals), methanol (for HPLC, VWR Chemicals), tetrahydrofuran (for HPLC, VWR Chemicals).

#### Synthesis of Re(bpy)(CO)_3_Cl

This complex was prepared with slight modifications to literature methods^9,28^. Re(CO)_5_Cl (0.3020 g, 0.83 mmol) was dissolved in 50 ml of hot toluene, then 2,2-bipyridine (0.130 g, 0.83 mmol) was added, the mixture was stirred and reflux for 1 h to get yellow product. Upon cooling, the product was filtered, washed with methanol for 3 times, dried under vacuum at 60 °C overnight and used without further purification. ^1^H NMR (δ, 400 MHz, DMSO-d_6_): 9.02 (d, 1H), 8.77 (d, 1H), 8.34(t, 1H), 7.76(t, 1H).

#### Synthesis of TpBpy

TpBpy was prepared according to literature methods with a little modification^74^. A Pyrex tube (o.d. × i.d. = 10 × 8 mm^2^ and length 25 cm)) was charged with triformylphloroglucinol (Tp) (21 mg, 0.10 mmol), 5,5′-diamino-2,2′-bipyridine (Bpy) (27.9 mg, 0.15 mmol), 0.5 Ml 1,4-dioxane, 0.5 Ml mesitylene, 0.1 Ml 6 M aqueous acetic acid. This mixture was sonicated for 20 min in order to get a homogeneous dispersion. The tube was flash-frozen in a liquid nitrogen bath, evacuated to an internal pressure of ca.0.15 mmHg and flame-sealed. The tube was placed in an oven at 120 °C for 5 days upon warming to room temperature to afford an orange-red precipitate. The precipitate was isolated by filtration over a medium glass frit and washed with anhydrous tetrahydrofuran (THF, 20.0 Ml). The product was immersed in anhydrous THF (20.0 Ml) for 8 h, during which the activation solvent was decanted and freshly replenished four times. The solvent was removed by filtration and the precipitate dried under vacuum at 60 °C overnight to afford TpBpy (42 mg, 86%).

#### Synthesis of Re-TpBpy

The process of synthesis Re-TpBpy was similar to that of Re-Bpy. Re(CO)_5_Cl (10 mg, 0.025 mmol) were dispersed in 10 Ml hot toluene, then TpBpy (25 mg) was added, the mixture was refluxed 40 min while stirring. The orange products were filtered, washed with methanol for 3 times, dried under vacuum at 60 °C overnight, and used without further purification.

#### Photocatalytic reduction of CO_2_

The method of photocatalytic reduction of CO_2_ was carried out according to literature methods with a little modification. Re-TpBpy (1 mg) was dispersed in 3 Ml of CH_3_CN, and 0.2 Ml of TEOA (triethanolamine) in 11 Ml septum-sealed glass vials^28^. The mixture was purged with Ar for 5 min and CO_2_ for 15 min first, then irradiated by a LED lamp with 520 nm and 440 wavelengths for 8 h and kept stirring during the photocatalytic reaction. The amount of CO generated was quantified using Shimadzu gas chromatography (GC-2010) by analyzing 0.5 Μl of the headspace. The cyclic photocatalysis experiment of Re-TpBpy under 440 nm excitation was carried out using the catalyst recovered in the previous reaction through centrifugation and drying under vacuum. As shown in Supplementary Fig. 16, the Re-TpBpy sample reveals a similar CO generation rate across three runs, indicating that the Re-TpBpy with considerable stability under 440 nm irradiation. Then, the photochemical experiment in the absence of CO_2_ was carried out (Supplementary Fig. 15). It is found that the CO generation rate is 1.9 μmol g^−1^ h^−1^, which is much lower than that with the existence of CO_2_ (19.6 μmol g^−1^ h^−1^). We also measured the adsorbents from the Re-TpBpy surface by mass spectrometry (OmniStarTM, Pfeiffer Vacuum, Germany). As presented in Supplementary Fig. 14, after Re-TpBpy sample was treated at 120 °C under the Ar atmosphere, there are only signals indexed to H_2_O (*m/z* = 18) and CO_2_ (*m/z* = 44) detected in mass spectra. It is suggested that there are only H_2_O and CO_2_ adsorption on the Re-TpBpy surface.

### Computational methods

To investigate the relationship of the optical properties with molecular structures and electronic structures, we used a triformylphloroglucinol (Tp) terminated bipyridine (Bpy) molecular fragment (Supplementary Fig. 12) to represent the COFs structure. An implicit solvent model was used to reflect the solvation environment, and implemented using SMD solvation model^75^ in Gaussian 16 package. Considering the transition metal complex in the fragment, M06-L^20,53–55,76,77^ was selected as the functional and def 2-TZVP^56,57^ was selected as the basis set for DFT calculations. Water and *n*-propanol parameters were used to represent the solvents in the SMD models^78^.

For the time-dependent density-functional theory (TD-DFT) calculations, we used the vertical electronic excitation approach to investigate the photo-induced charge-transfer behavior of Re-TpBpy. The ground state structures were taken from the above-mentioned structural optimization results. TD-DFT calculations were performed at the M06-L/def 2-TZVP level of theory with SMD solvation model, and the number of calculated excitation state was set to 20.

The crystalline structures of TpBpy were calculated using density-functional theory^79,80^ (DFT) implemented in the CASTEP^81^ module of Materials Studio 7.0. The generalized gradient approximation (GGA) in the form of Perdew–Burke–Ernzerhof (PBE)^82^ was selected as the exchange-correlation functional. Grimme dispersion correction^83,84^ was employed in all calculations to describe van der Waals (vdW) and π-stacking interactions. The lattice dimensions were optimized simultaneously with the geometry. A plane wave energy cutoff of 830 eV and the Monkhorst-Pack *k*-point grid of 1 × 1 × 4 were used. The convergence criteria for energy, force, stress, and displacement are 5 × 10^−6^ eV/atom, 0.01 eV/Å, 0.02 GPa and 5 × 10^−4^ Å, respectively. All simulation works were performed using the computing resources at National Supercomputing Center in Shenzhen.

The UV-vis absorption spectra and electron excitations were analyzed using the Multiwfn program^85^.

### Characterization

Powder X-ray diffraction (PXRD) data were collected by using Rigaku Miniflex600 at room temperature with Cu Kα1 source (*λ* = 1.5418 Å) over the range of 2*θ* = 3.0–40.0° with a step size of 0.02° and a counting time of 1 s per step. Fourier-Transformed Infrared Spectroscopy (FT-IR) data were obtained by using ALPHA P FT-IR spectrometer (Bruker). The sample material just has to be brought into contact with the measurement interface. X-ray Photoelectron Spectroscopy (XPS) data were got by using XPS-Thermo Scientific with Al Kα (1486 eV) as the excitation X-ray source. The pressure of the analysis chamber was maintained at 2 × 10^−10^ mbar during measurement. The sample material was prepared by dispersing it in ethanol and then dripping it onto a silicon wafer sprayed with 39.7 nm gold by Quorum Coater, then dried in air. The peak of C 1*s* at about 284.8 eV was used to calibrate the energy scale. The XPS data were performed to analyze the valence band maximum (VBM) position corresponding to the Fermi level and compositions of samples. The absorption spectra were measured in a UV-Vis absorption spectrophotometer from Agilent Technologies (Santa Clara, USA). Photoluminescence (PL) was performed via Spex Fluorolog 1681 standard spectrofluorometer. Time-correlated single-photon counting (TCSPC, Picoharp) was performed triggered externally at 2.5 MHz to excite the sample at 438 nm. The emitted photons were detected by a fast avalanche photodiode (SPAD, Micro Photon Device) with response time <50 ps after passing through a 470 nm long band pass filter. The TRPL measurements were also performed by a streak camera setup (Hamamatsu) using a pulsed fs-laser for excitation (wavelength: 400 nm; frequency: 80 MHz; pulse duration: 150 fs). It is important to note that during all photophysical measurements the sample material was dispersed in Nafion (5% w/w in water and 1-propanol) except Tp was dispersed in acetonitrile.

Scanning electron microscopy (SEM) images were obtained by using AFEG 250 Analytical ESEM at an accelerating voltage of 20.0 kV shown in Supplementary Fig. 4. Transmission electron microscopy (TEM) images and Energy dispersive X-ray (EDX) mapping images were obtained with a Tecnai G2 T20 TEM shown in Supplementary Fig. 5 and Supplementary Fig. 6, respectively.

Nuclear magnetic resonance (NMR) spectroscopy was acquired on Bruker AVANCE 400 MHz spectrometer with a 5 mm CryoProbe Prodigy using ~1 mg sample dissolved in 2 mL of deuterated dimethyl sulfoxide (DMSO-d_6_). The ^13^C solid-state MAS NMR spectra were recorded on a 14.1 T AVANCEIII HD spectrometer (Bruker) equipped with a 4 mm CP/MAS probe (Bruker). The spectra were recorded with a π/2 pulse of 3.75 μs, an interscan delay of 45 s and *υ*_R_ = 7KHz. High-power 1H decoupling was applied during acquisition. Chemical shifts are reported relative to TMS (*d*_iso_ = 0 ppm) using a secondary reference of adamantane (*d*_iso_ = 38.48 ppm). 2000 transient scans were recorded for each spectrum.

#### Cyclic voltammetry (CV)

The cyclic voltammetry (CV) experiment was carried out on an Autolab PGSTAT12 instrument (Eco Chemie, Switzerland) with three-electrode systems: glassy carbon working electrode, a platinum counter electrode, and an Ag/AgCl reference electrode. CH_3_CN solution having tetrabutylammonium hexafluorophosphate (NBu_4_PF_6_) (0.1 mol L^−1^) as electrolyte at a scan rate of 20 mV s^−1^ and 0.01 M ferrocene (Fc/Fc^+^) as internal standard at room temperature. For Tp, Bpy, and Re-Bpy, the electrodes were modified in 1 mM CH_3_CN solution. For TpBpy and Re-TpBpy, the working electrodes were prepared as follows: 1 mg TpBpy or Re-TpBpy was mixed with 0.5 mL 1 mL ethanol and 30 μL Nafion (10 wt% in water) sonicated for 2 h to form a homogeneous slurry. Afterward, the slurry was coated on a glassy carbon working electrode and then dried in the air. All electrolytes were purged with argon gas for at least 30 min and an argon atmosphere was maintained during electrochemical measurements. All potentials are referred to ferrocene (Fc/Fc^+^). The voltammograms are shown in Supplementary Fig. 4. The highest occupied molecular orbital (HOMO) and lowest unoccupied molecular orbital (LUMO) energy levels were calculated by combining both absorption spectroscopy and CV. The LUMO energy levels were calculated from the first reduction onset potential using as reported equation *E*_LUMO_ = − (*E*_red/onset_ + 4.4) eV and the HOMO energy levels were determined by the optical bandgap (*E*_g_^opt^ = 1240/*λ*_edge_) plus LUMO energy levels.

#### Transient absorption (TA) spectroscopy measurements

Time-resolved experiments were carried out on laser-based spectroscopy, with laser powers equating to less than one photon absorption per particle. Samples for transient absorption experiments were kept in dark between each measurement. A Coherent Legend Ti: Sapphire amplifier (800 nm, 100 fs pulse length, 3 kHz repetition rate) was used. The output is split to pump and probe beams. Excitation pulses at the wavelength of 450 nm were acquired using an optical parametric amplifier (Topas C, Light Conversion). The probe pulses (a broad supercontinuum spectrum) were generated from the 800-nm pulses in a CaF_2_ crystal and split by a beam splitter into a probe pulse and a reference pulse. The probe pulse and the reference pulse were dispersed in a spectrograph and detected by a diode array. Instrumental response time is ∼100 fs.

#### Transient Mid-IR absorption spectroscopy

A frequency doubled Q-switched Nd:YAG laser (Quanta-Ray ProSeries, Spectra-Physics) was employed to obtain 400 and 530 nm pump light, 10 mJ/pulse with a fwhm of 10 ns. The 400 and 530 nm pump light was used through the MOPO crystal to pump the sample. Probing was done with the continuous wave quantum cascade (QC) IR laser with a tuning capability between 1960 and 2150 cm^−1^ (Daylight Solutions). For IR detection, a liquid nitrogen-cooled mercurycadmium-telluride (MCT) detector (KMPV10^−1^J^2^, Kolmar Technologies, Inc.) was used. The IR probe light was overlapped with the pump beam in a quasi-co-linear arrangement at 25° angle. Transient absorption traces were acquired with a Tektronix TDS 3052 500 MHz (5 GS/s) oscilloscope in connection with the L900 software (Edinburgh Instruments) and processed using Origin 9 software^86,87^. Samples were kept in a modified Omni cell (Specac) with O-ring sealed CaF_2_ windows and a path lenth of 1 mm. All samples were prepared in an Ar-filled glove box (Unilab, MBraun), and Nafion (5% w/w in water and 1-propanol) was used as solvent in all experiments. All acids were dried overnight under vacuum prior to use.

### Data analysis

Transient absorption data analysis was performed with the singular value decomposition (SVD) in combination with Global-fit system of Surface Xplorer software package (https://ultrafastsystems.com/surface-xplorer-data-analysis-software/). The two-dimensional (2D) transient absorption data is an *n* × *m* matrix composed of time points (*n*) and wavelengths (*m*). The principle of the SVD fitting is to obtain several main spectral components from the original data through mathematical processing, and then obtain the spectrum and time dynamics curves of transient species by analyzing several principal components, which greatly reduces workload and noise^88,89^. The SVD procedure results in a basis set of eigenvectors whose matrix product A is given by:

1 $$A={{USV}}^{T}$$

where *U* and *V* are column vectors (orthogonal normalization matrix), *S* is a diagonal matrix. *U* is the spectral componets, *V* is the time dynamics componets, *S* is the singular value (nonnegative elements). The real data matrix of SVD usually contains several main components with large s value and a large number of components with s value close to zero. These small components are usually considered as noise, we only analyze SVD components with large s values, which can simplify the analysis process. The selected principal kinetic traces are globally fitted to a multi-exponential decay law convoluted with the Gaussian instrument response function using the nonlinear Marquardt algorithm, and the timescale corresponding to each decay process and the dynamic behavior curve of each component are obtained^90,91^.

### Calculation details of the apparent quantum yield (AQY)

The energy of one photon (*E*_photon_) with the wavelength of *λ*_inc_ (nm) is calculated by the following equation:

2 $${E}_{{{{{{\rm{photon}}}}}}}=\frac{hc}{{\lambda }_{{{{{{\rm{inc}}}}}}}}$$

where *h* (J·s) is Planck’s constant, *c* (m·s^−1^) is the speed of light and *λ*_inc_ (m) is the wavelength of the incident monochromatic light.

The total energy of the incident monochromatic light (*E*_total_) is calculated by the following equation:

3 $${E}_{{{{{{{\mathrm{total}}}}}}}}={I}_{{{{{{{\mathrm{inc}}}}}}}}{St}$$

where *I*_inc_ (W·m^−2^) is the power density of the incident monochromatic light, *S* (m^2^) is the irradiation area and *t* (s) is the duration of the incident light exposure.

According to the Beer–Lambert law:

4 $$A={\log_{10}}\frac{{I}_{{{{{{{\mathrm{inc}}}}}}}}}{{I}_{{{{{{{\mathrm{tra}}}}}}}}}=\varepsilon {cL}$$

Where *A* is the measured absorbance, *I*_inc_ (W·m^−2^) is the intensity of the incident light at a given wavelength, *I*_tra_ (W·m^−2^) is the transmitted intensity, *L* (m) the path length through the sample, and *c* (mol·L^−1^) the concentration of the absorbing species.

Then the power density of absorbed monochromatic light (*I*_a_) by catalysts is:

5 $${I}_{a}={I}_{{{{{{{\mathrm{inc}}}}}}}}-{I}_{{{{{{{\mathrm{tra}}}}}}}}$$

Then the power density of absorbed monochromatic light (*I*_a_) by catalysts is:

6 $${E}_{a}={E}_{{{{{{{\mathrm{total}}}}}}}}-{E}_{{{{{{{\mathrm{tra}}}}}}}}=({I}_{{{{{{{\mathrm{inc}}}}}}}}-{I}_{{{{{{{\mathrm{tra}}}}}}}}){St}$$

The number of absorbed photons (*N*_photon_) by catalysts can be obtained through the following equation:

7 $${N}_{{{{{{{\mathrm{photon}}}}}}}}=\frac{{E}_{a}}{{E}_{{{{{{{\mathrm{photon}}}}}}}}}=\frac{{({I}_{{{{{{{\mathrm{inc}}}}}}}}-{I}_{{{{{{{\mathrm{tra}}}}}}}}){St}\lambda }_{{{{{{{\mathrm{inc}}}}}}}}}{{hc}}$$

The moles of incident photons (*n*_photon_) absorbed by catalysts can be obtained through the following equation:

8 $${n}_{{{{{{{\mathrm{photon}}}}}}}}=\frac{{N}_{{{{{{{\mathrm{photon}}}}}}}}}{{N}_{{{{{{\mathrm{A}}}}}}}}=\frac{{({I}_{{{{{{{\mathrm{inc}}}}}}}}-{I}_{{{{{{{\mathrm{tra}}}}}}}}){St}\lambda }_{{{{{{{\mathrm{inc}}}}}}}}}{{hc}{N}_{{{{{{\mathrm{A}}}}}}}}$$

Where *N*_A_ (mol^−1^) is the Avogadro constant.

Finally, The AQY is calculated by the number of molecules Nmol undergoing an event (conversion of reactants or formation of products) relative to the number of photon Nph absorbed by the photocatalyst in the following expression:

9 $${\varphi }_{{{{{{{\mathrm{AQY}}}}}}}}=\frac{{n}_{{{{{{{\mathrm{mol}}}}}}}}\left({{{mol}}}\,{{{s}}}^{-1}\right)}{{n}_{{ph}}\left({{{einstein}}}\,{{{s}}}^{-1}\right)}$$

## Supplementary information

Supplementary Information

## Data Availability

The raw images of molecular structures in Fig. 1a, Supplementary Figs. 1, 2, 12, 13; SEM images in Supplementary Fig. 6; HR-TEM images Supplementary Fig. 7; and EDX mapping images in Supplementary Fig. 8 are available on a reasonable request from the corresponding author using the contact email: kzheng@kemi.dtu.dk, for their large size reason. Source data are provided with this paper.

## Source data

Source Data
