# Supplementary material for: Ultrafast charge transfer dynamics in 2D covalent organic frameworks/Re-complex hybrid photocatalyst
Source: Nat Commun. 2022 Feb 11;13:845. doi: 10.1038/s41467-022-28409-2 (PMC8837612; doi:10.1038/s41467-022-28409-2)
Supplement: Supplementary file 1 — Supplementary Information [file 41467_2022_28409_MOESM1_ESM.pdf]

Supplementary Information

Contents

Supplementary Figures 2
Supplementary Tables 11
Supplementary References 33

Supplementary Figures

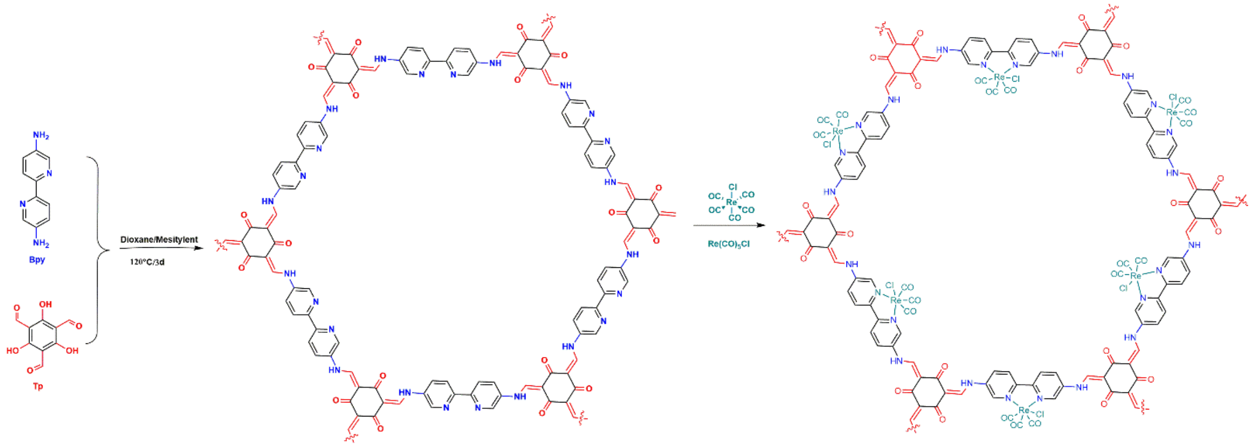

Supplementary Figure 1: Re-COF Synthesis. Synthesis scheme for TpBpy and Re-TpBpy.

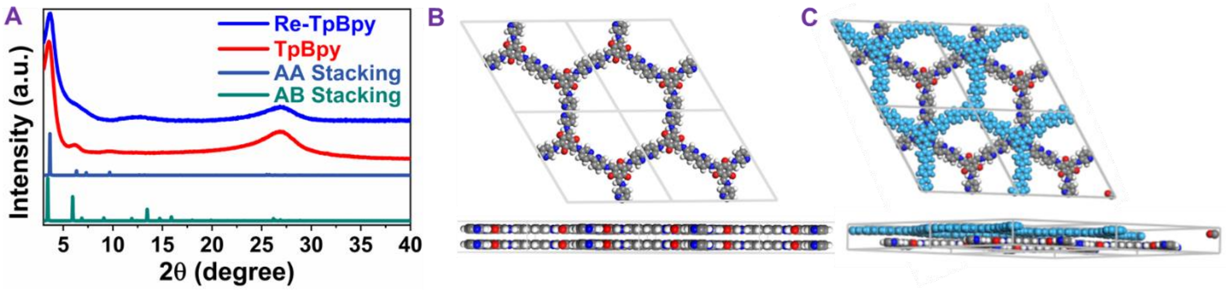

Supplementary Figure 2: XRD characterization of the COF. A) PXRD of TpBpy (red curve), Re-TpBpy (blue curve), B) simulated AA stacking mode (Dark blue curve), and C) simulated AB stacking mode (Dark green curve). **Source data are provided as a Source Data file.** The powder X-ray diffraction (PXRD) patterns of both TpBpy and Re-TpBpy match well with the simulated AA stacking structure of COFs in the hexagonal space group (P6). The pronounced peak at 2θ of 3.6° of both samples can be assigned to the (100) plane of the TpBpy, which demonstrates that the crystalline structure of TpBpy is unchanged after Re-complex incorporation. The broad peak between 2θ = 25.1°-28.1° is attributed to the π-π stacking interlayers along the (001) plane[1].

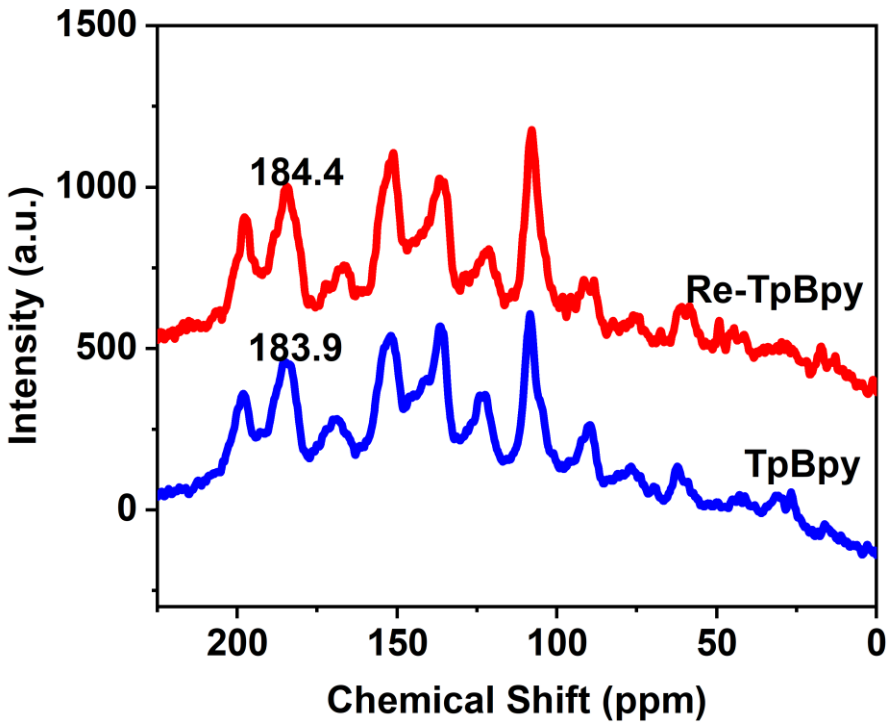

Supplementary Figure 3: Solid-state NMR characterization. Solid-state ^{13}C NMR spectra of TpBpy and Re-TpBpy. **Source data are provided as a Source Data file.** A slight shift in the area of 184 ppm compared to the Re-TpBpy with pure TpBpy, which is due to the weak nonbonding interactions with the pyridinic nitrogen of the bipyridinic. This observation confirms that the Re-complex was incorporated in the TpBpy. A similar shift and assignment have also been evidenced in Ni-TpBpy with a similar structural configuration[2].

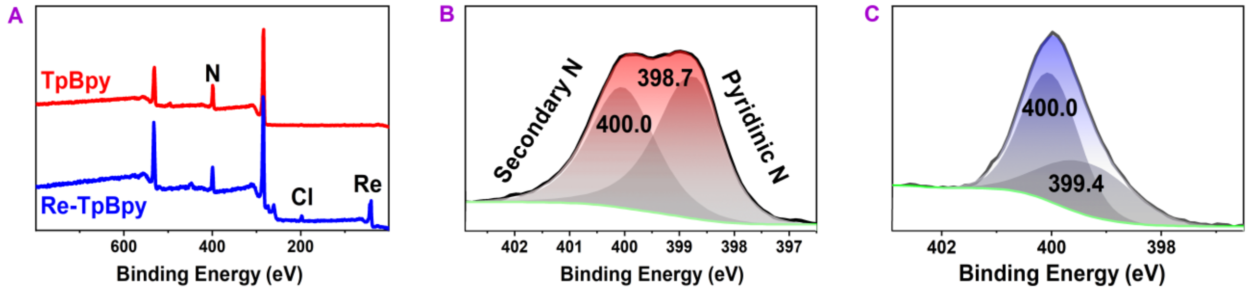

Supplementary Figure 4: XPS characterization. A) overview XPS spectrum of TpBpy and Re-TpBpy, (B) and (C) correspond to the XPS N 1s core level spectra of TpBpy and Re-TpBpy, respectively. The overview spectrum of Re-TpBpy displays the existence of Re and Cl elements in addition to C, O, N in pristine TpBpy, suggesting the actual incorporation of the Re-complex in the host COF. More importantly (Supplementary fig.4a), the two N 1S bands observed at 400.03 eV and 398.71 eV can be ascribed to the secondary nitrogen and pyridinic nitrogen in TpBpy, respectively[3,4]. In Re-TpBpy, the pyridinic nitrogen

band is shifted to higher binding every (i.e. from 398.71 eV to 399.39 eV), while the peak position of the secondary nitrogen (Supplementary fig.4b, c) remains unchanged. This indicates that the Re-complex is anchored to the TpBpy only through its bipyridinic units[5]. **Source data are provided as a Source Data file.**

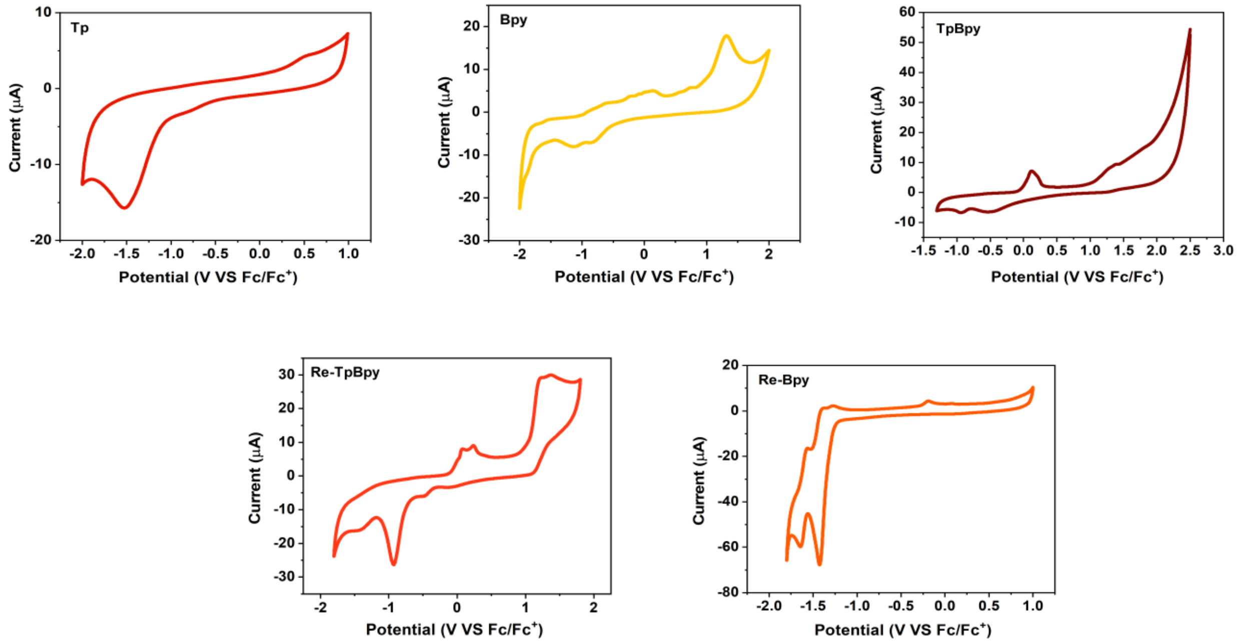

Supplementary Figure 5: Electrochemistry characterization. Cyclic voltammetry curves of samples. **Source data are provided as a Source Data file.**

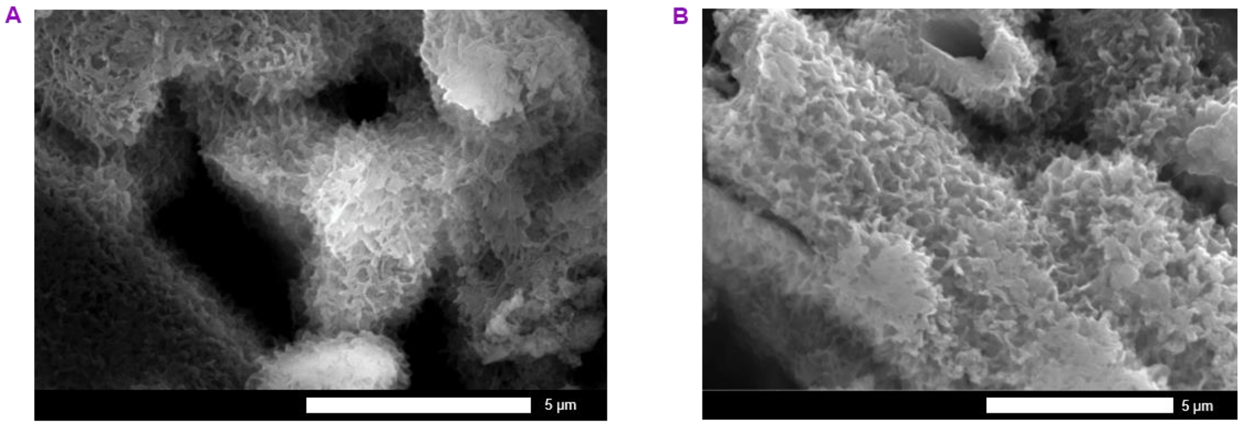

Supplementary Figure 6: SEM characterization. SEM image of TpBpy (A) and Re-TpBpy (B).

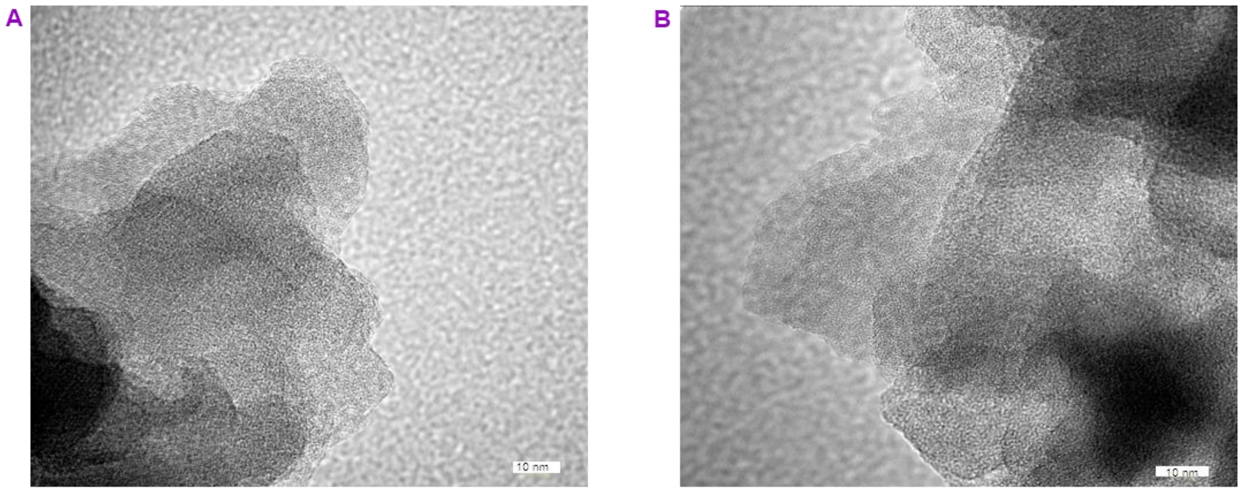

Supplementary Figure 7: HR-TEM characterization. HR-TEM image of TpBpy (A) and Re-TpBpy (B).

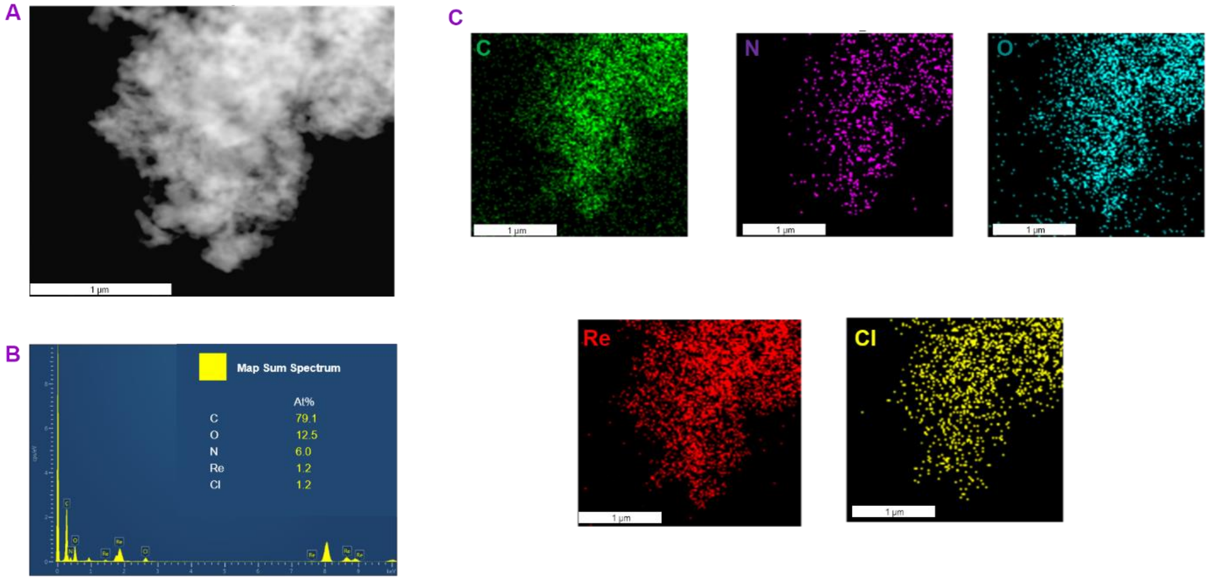

Supplementary Figure 8: EDX characterization. TEM image of Re-TpBpy (A), EDX Spectrum of Re-TpBpy (B). The element analysis results of Re-TpBpy (C). **Source data are provided as a Source Data file.** Compared the SEM image of Re-TpBpy with the original TpBpy, the Re-modified TpBpy did not observe any significant changes in morphology (see see supplementary fig. 6). In addition, the TEM image did not show the presence of any metal oxide particles in the COF matrix (see supplementary fig. 7). However, TEM-EDX mapping reveals a uniform distribution of Re content in Re-TpBpy (see supplementary fig. 8).

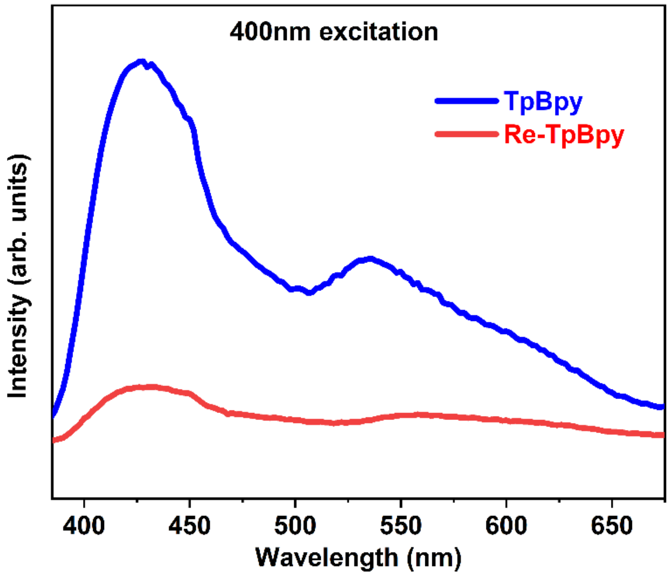

Supplementary Figure 9: Steady-state PL measurement at 400 nm excitation. Emission spectra of TpBpy and Re-TpBpy calibrated by the absorption at the 400 nm excitation wavelength. **Source data are provided as a Source Data file.**

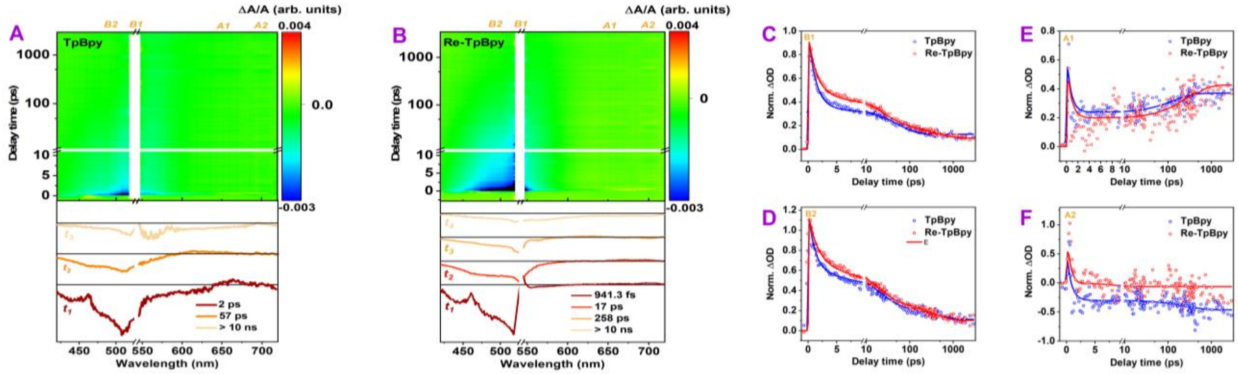

Supplementary Figure 10: TA measurement at band edge excitation with high excitation intensity. TA spectra under 530 nm excitation at the fluence of 1×10^{14} ph/cm^2 and the respective SVD fittings of TpBpy (A), Re-TpBpy (B). TA kinetics of two samples at various emission wavelengths of representing B1 (C), B2 (D), A1 (E), A2 (F). All spectra are recorded in Nafion (5% w/w in water and 1-propanol). **Source data are provided as a Source Data file.**

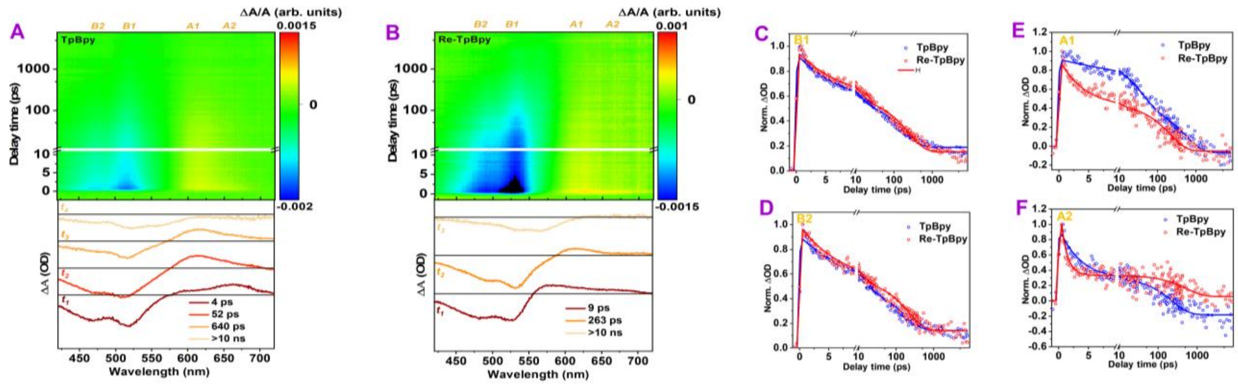

Supplementary Figure 11: TA measurement at high excitation energy with high excitation intensity. TA spectra under 400 nm excitation at the fluence of 1×10^{14} ph/cm^2 and the respective SVD fittings of TpBpy (A), Re-TpBpy (B). TA kinetics of two samples at various emission wavelengths of representing B1 (C), B2 (D), A1 (E), A2 (F). All spectra are recorded in Nafion (5% w/w in water and 1-propanol). **Source data are provided as a Source Data file.**

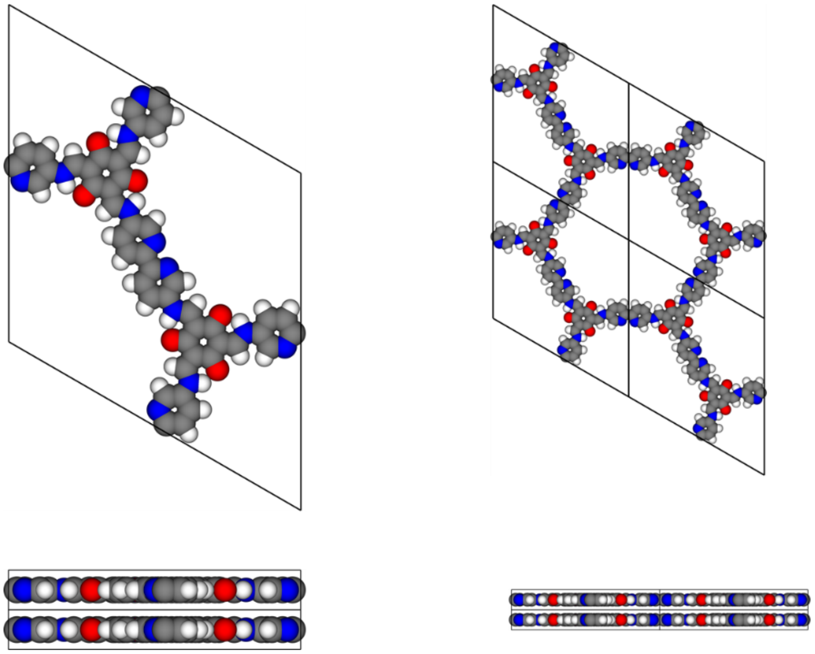

Supplementary Figure 12: 3D view of the molecular structure for TpBpy. Top-view and side-view of simulated AA stacking mode of TpBpy, crystalline cell (left) and periodic crystal structure (right). TpBpy exhibit the symmetry of P6 in its lattice.

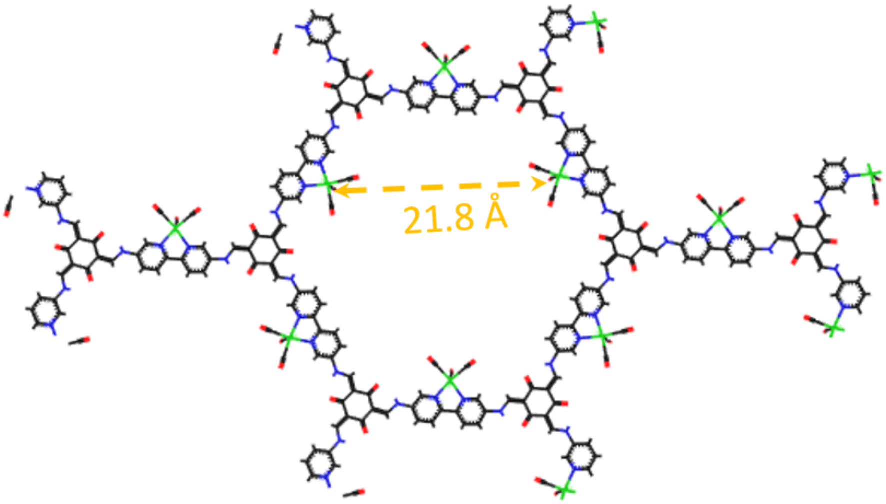

Supplementary Figure 13: Calculation of the Re-to-Re spacing in Re-TpBpy. Top view of crystalline Structures of Re-TpBpy. The pore size is 21.8 Å according to the simulation.

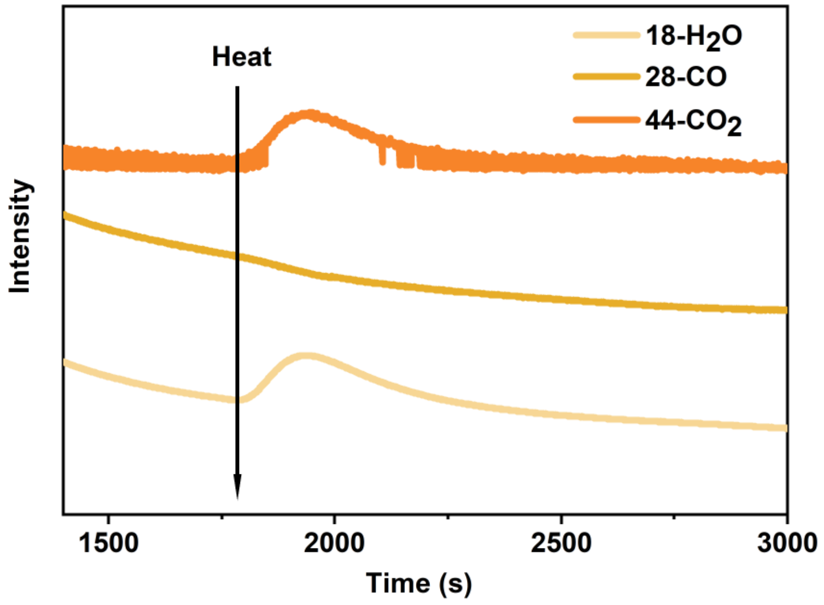

Supplementary Figure 14: Thermal stability of the Re-COF. The mass spectra of Re-TpBpy treated at 120 °C under the Ar atmosphere. **Source data are provided as a Source Data file.**

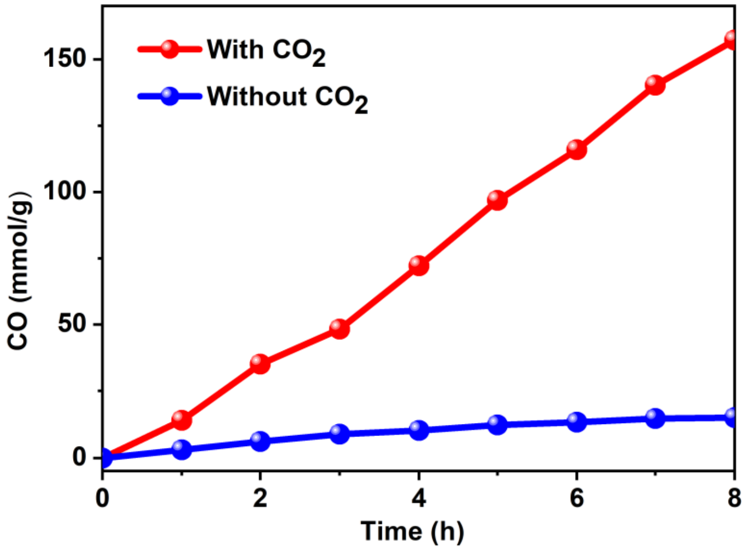

Supplementary Figure 15: Identification of the catalytic products. Production of CO in the photocatalytic conversion of CO_2 with or without CO_2. **Source data are provided as a Source Data file.**

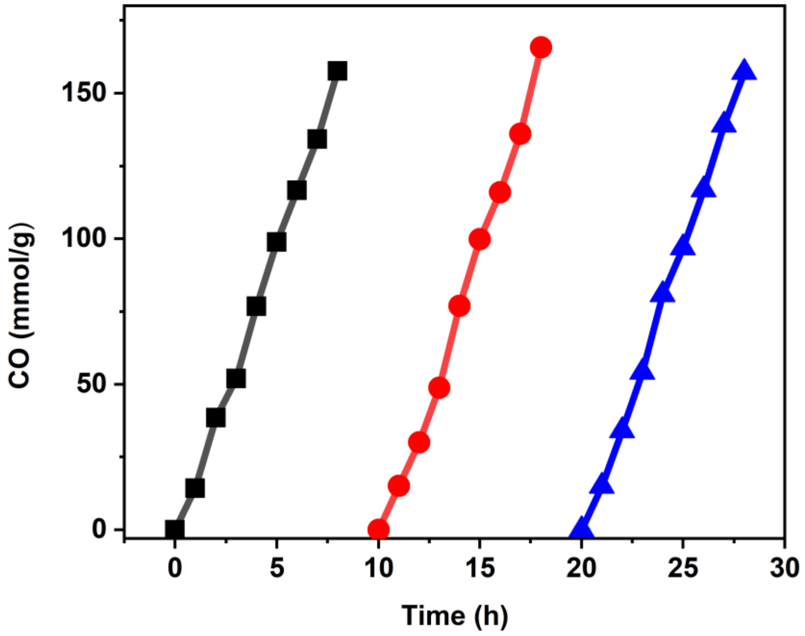

Supplementary Figure 16: Reproducibility of catalytic reaction. Recycle photocatalytic CO_2 reduction experiments over Re-TpBpy under 440 nm excitation. **Source data are provided as a Source Data file.**

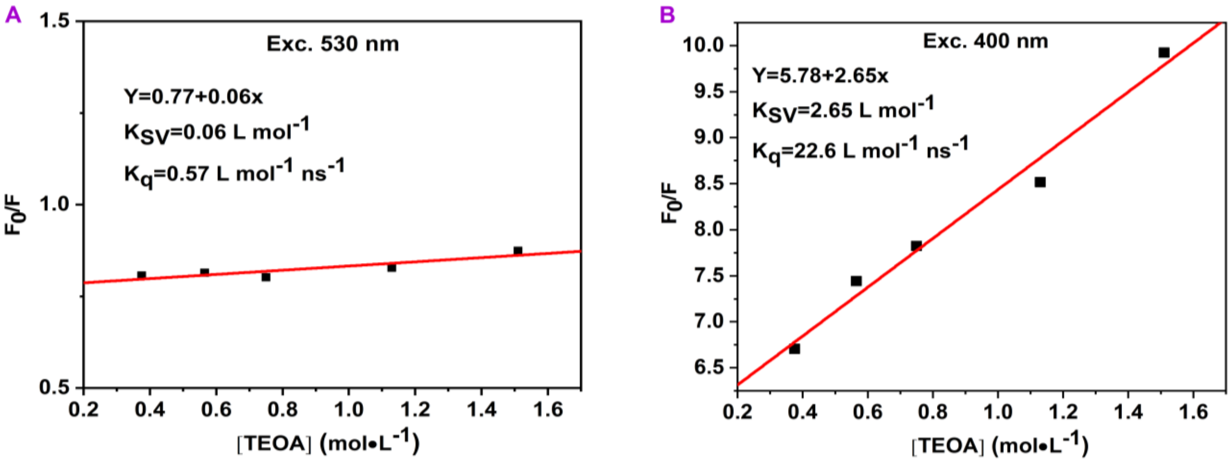

Supplementary Figure 17: PL quenching experiment. Stern-Volmer plot of Re-TpBpy (excited under 530 nm (A) and 400 nm (B)) describes the fluorescence quenching intensity vs. the TEAO concentration over the range 0.37–1.51 mol L^{-1}. **Source data are provided as a Source Data file.**

Supplementary Tables

Supplementary Table 1: Optical and electrochemical properties of samples.

Sample	$E_{red,onset}$ (V)	λ_{edge} (nm)	E_g^{opt}	HOMO (eV)	LUMO (eV)
Tp	-1.05	403.8	3.07	-0.28	-3.35
Bpy	-0.48	458.8	2.70	-1.22	-3.92
TpBpy	0.15	558.4	2.22	-2.33	-4.55
Re-TpBpy	-0.72	567.6	2.18	-1.50	-3.68
Re-Bpy	-1.29	435.0	2.85	-0.26	-3.11

Supplementary Table 2: Calculated DFT energy levels of TpBpy fragment.

Orbital Name	Orbital Number	Energy (eV)	iso-surface images
LUMO+12	240	-0.96886231	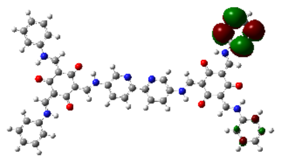
LUMO+11	239	-0.969700086	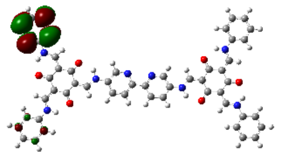
LUMO+10	238	-0.972883907	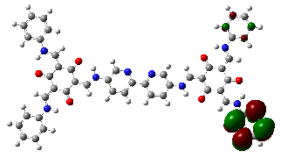
LUMO+9	237	-0.973715102	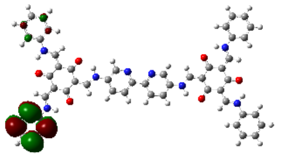
LUMO+8	236	-1.068732822	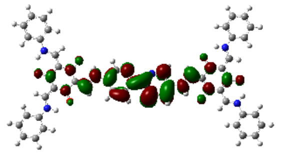
LUMO+7	235	-1.333214011	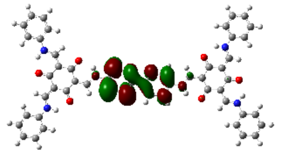
LUMO+6	234	-1.621848404	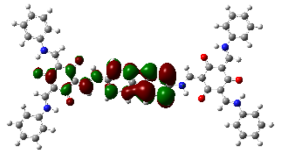
LUMO+5	233	-1.995218628	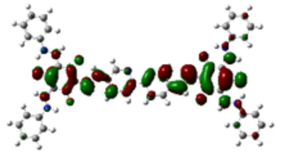

LUMO+4	232	-2.034934733	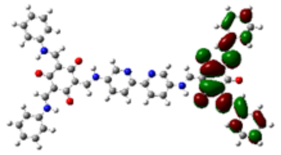
LUMO+3	231	-2.035340048	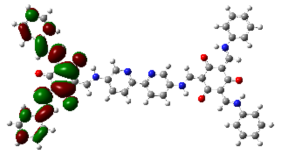
LUMO+2	230	-2.237486536	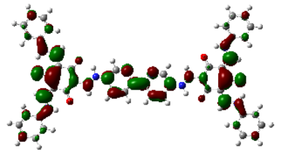
LUMO+1	229	-2.576204276	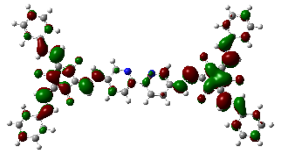
LUMO	228	-2.799618969	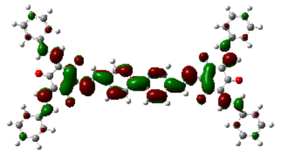
HOMO	227	-5.045478166	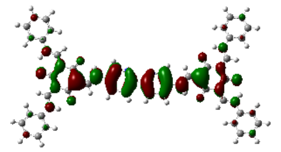
HOMO-1	226	-5.172556295	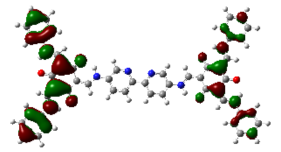
HOMO-2	225	-5.172573899	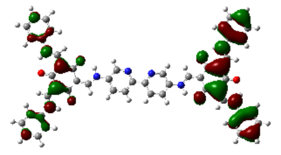

HOMO-3	224	-5.365218278	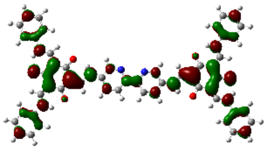
HOMO-4	223	-5.670073594	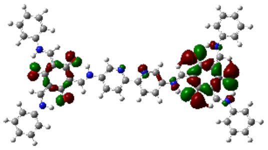
HOMO-5	222	-5.676649	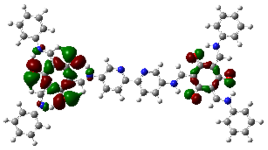
HOMO-6	221	-5.718600616	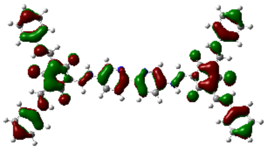
HOMO-7	220	-5.940760198	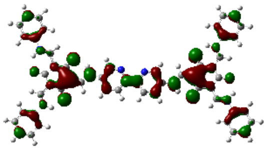
HOMO-8	219	-6.053834645	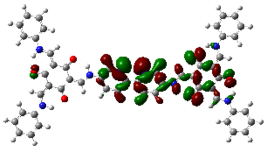
HOMO-9	218	-6.200828562	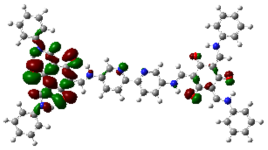
HOMO-10	217	-6.205291056	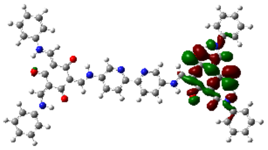

HOMO-11	216	-6.22438551	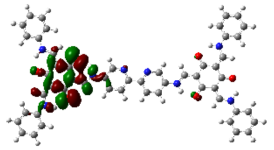
HOMO-12	215	-6.279711603	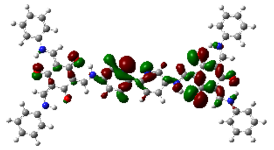

Supplementary Table 3: Calculated DFT energy levels of Re-TpBpy fragment.

Orbital Name	Orbital Number	Energy (eV)	iso-surface images
LUMO+12	277	-0.98413959	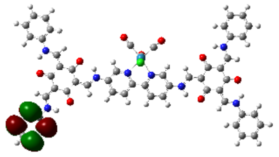
LUMO+11	276	-0.986257352	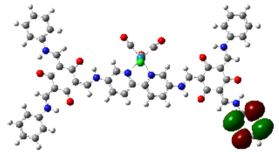
LUMO+10	275	-1.078184819	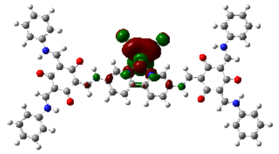
LUMO+9	274	-1.283727355	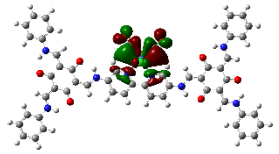
LUMO+8	273	-1.402089054	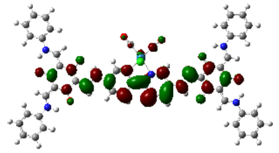
LUMO+7	272	-1.867455072	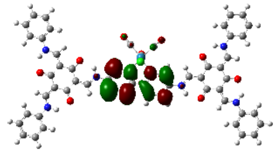
LUMO+6	271	-1.993478502	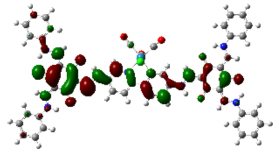
LUMO+5	270	-2.067937563	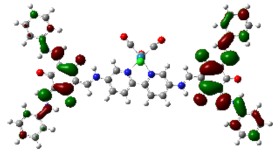

LUMO+4	269	-2.068107788	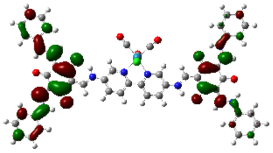
LUMO+3	268	-2.196090793	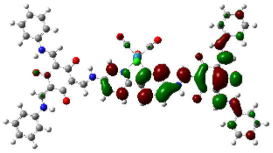
LUMO+2	267	-2.455782405	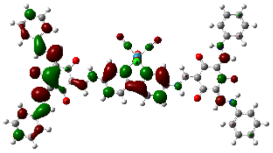
LUMO+1	266	-2.655408515	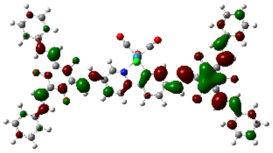
LUMO	265	-3.092359577	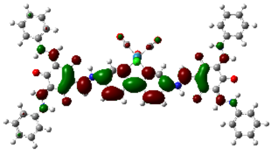
HOMO	264	-5.197598202	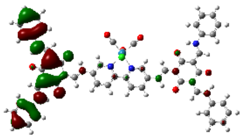
HOMO-1	263	-5.19983685	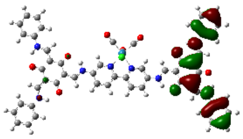
HOMO-2	262	-5.206329891	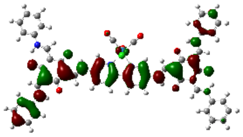

HOMO-3	261	-5.429726984	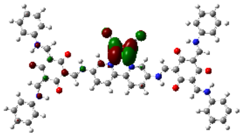
HOMO-4	260	-5.450935274	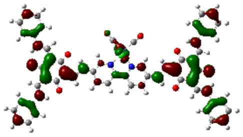
HOMO-5	259	-5.529614132	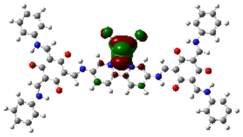
HOMO-6	258	-5.724384074	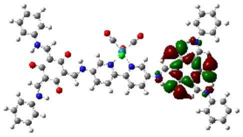
HOMO-7	257	-5.72505861	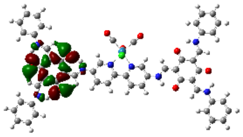
HOMO-8	256	-5.811185661	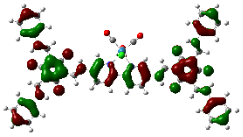
HOMO-9	255	-5.893560943	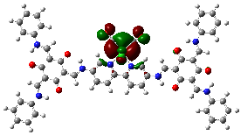
HOMO-10	254	-6.051646689	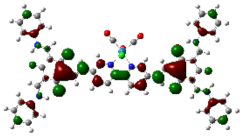

HOMO-11	253	-6.242005536	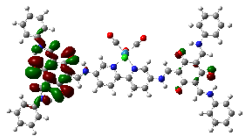
HOMO-12	252	-6.242635938	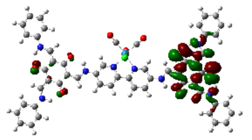

Supplementary Table 4: Atomistic coordinates of TpBpy-AA optimized by using DFT method.

Space group: *P-6;*

$a = b = 29.8346$ Å, $c = 3.6445$ Å;

$\alpha = \beta = 90^\circ$, $\gamma = 120^\circ$.

**Atom**	***x/a***	***y/b***	***z/c***
C	0.49681	-0.53229	-0.5
C	0.46766	-0.5868	-0.5
C	0.49265	-0.61542	-0.5
C	0.54689	-0.58948	-0.5
C	0.57318	-0.53496	-0.5
N	0.54931	-0.50721	-0.5
N	0.57174	-0.61881	-0.5
C	0.62304	-0.60123	-0.5
C	0.6435	-0.63404	-0.5
C	0.60977	-0.6902	-0.5
O	0.72841	-0.56111	-0.5
H	0.55054	-0.65954	-0.5
C	0.47198	-0.49978	-0.5
N	0.50378	-0.44766	-0.5
C	0.48261	-0.41752	-0.5
C	0.42891	-0.43621	-0.5
C	0.39593	-0.48999	-0.5
C	0.41777	-0.52157	-0.5
N	0.41185	-0.40019	-0.5
C	0.36251	-0.41033	-0.5
C	0.34919	-0.37194	-0.5
C	0.38891	-0.31726	-0.5
O	0.26019	-0.43642	-0.5
H	0.43816	-0.36018	-0.5
H	0.42552	-0.60725	-0.5
H	0.47074	-0.65766	-0.5
H	0.61548	-0.51266	-0.5
H	0.64985	-0.55959	-0.5
H	0.50933	-0.37562	-0.5
H	0.354	-0.5071	-0.5
H	0.39207	-0.56335	-0.5
H	0.33129	-0.45057	-0.5

Supplementary Table 5: Atomistic coordinates of TpBpy-AB optimized by using DFT method.

Space group: *P-6;*

a = *b* = 29.7647 Å, *c* = 6.1262 Å;

α = *β* = 90°, *γ* = *120*°.

**Atom**	***x/a***	***y/b***	***z/c***
C	1.82204	1.12635	0
C	1.79351	1.07167	0
C	1.8194	1.04367	0
C	1.87383	1.07047	0
C	1.89941	1.12511	0
N	1.87471	1.15217	0
N	1.89997	1.0422	0
C	1.9517	1.06154	0
C	1.97441	1.03057	0
C	1.94272	0.97402	0
O	2.05796	1.10623	0
H	1.87973	1.00149	0
C	1.79691	1.15875	0
N	1.82918	1.21098	0
C	1.80843	1.24156	0
C	1.75465	1.22328	0
C	1.72106	1.16939	0
C	1.74257	1.13726	0
N	1.73887	1.26022	0
C	1.69036	1.25213	0
C	1.6797	1.29263	0
C	1.72145	1.34657	0
O	1.58967	1.23136	0
H	1.7664	1.30001	0
H	1.75125	1.0507	0
H	1.79798	1.00133	0
H	1.94175	1.14814	0
H	1.97725	1.10351	0
H	1.83551	1.28349	0
H	1.67905	1.15264	0
H	1.71661	1.09539	0
H	1.65786	1.2124	0
C	0.48861	0.45891	0.5
C	0.45976	0.40421	0.5
C	0.4853	0.37582	0.5
C	0.53975	0.40232	0.5
C	0.56563	0.457	0.5
N	0.54126	0.48436	0.5
N	0.56586	0.37406	0.5

C	0.61768	0.39391	0.5
C	0.64066	0.36333	0.5
C	0.60949	0.30688	0.5
O	0.72361	0.43957	0.5
H	0.54596	0.33334	0.5
C	0.46399	0.49178	0.5
N	0.49656	0.54395	0.5
C	0.47619	0.5749	0.5
C	0.42251	0.55705	0.5
C	0.38866	0.50323	0.5
C	0.40973	0.47074	0.5
N	0.40686	0.59427	0.5
C	0.35811	0.5861	0.5
C	0.34694	0.62621	0.5
C	0.38843	0.68043	0.5
O	0.2568	0.56442	0.5
H	0.43442	0.63406	0.5
H	0.41749	0.38347	0.5
H	0.46354	0.33346	0.5
H	0.60796	0.47979	0.5
H	0.6429	0.43583	0.5
H	0.50356	0.61677	0.5
H	0.34671	0.48683	0.5
H	0.38352	0.42892	0.5
H	0.32578	0.54626	0.5

Supplementary Table 6: Atomistic coordinates of TpBp-n_propanol.

Space group: *P-6;*

a = *b* = 29.7171 Å, *c* = 3.6389 Å;

α = *β* = 90˚, *γ* = 120˚.

Atom	*x (Å)*	*y (Å)*	*z (Å)*
C	12.82607	8.28556	2.55511
C	13.45496	7.06020	2.78772
C	14.82914	6.98689	2.77993
C	15.57025	8.13988	2.53708
C	14.86423	9.32363	2.31420
N	13.54423	9.39525	2.32468
N	16.95759	8.05678	2.53155
C	17.82925	9.04245	2.34057
C	19.20221	8.90143	2.34951
C	19.83544	7.61748	2.57592
O	19.41644	11.21299	1.93258
H	17.39390	7.14591	2.69329
C	11.35888	8.41665	2.55693
N	10.84719	9.64553	2.72987
C	9.53595	9.79424	2.73398
C	8.63153	8.74398	2.56386
C	9.15480	7.46825	2.37948
C	10.52345	7.31106	2.37884
N	7.27541	9.04021	2.58461
C	6.26091	8.19419	2.43942
C	4.92571	8.54401	2.45996
C	4.50097	9.91679	2.65064
O	4.35359	6.28294	2.11228
H	6.98782	10.01278	2.71924
H	12.87726	6.17008	2.99502
H	15.33747	6.04880	2.96769
H	15.38984	10.25146	2.11454
H	17.43134	10.03433	2.16596
H	9.15278	10.80138	2.88183
H	8.51277	6.61066	2.23157
H	10.93942	6.32606	2.21804
H	6.50186	7.14816	2.29282
C	25.17733	2.46452	3.50020
C	25.86933	3.63980	3.24436
C	25.18371	4.82098	3.02469
C	23.78974	4.83829	3.05894
C	23.09088	3.66029	3.31675

C	23.78958	2.48538	3.53452
N	23.15734	6.06423	2.82988
C	21.85689	6.31513	2.79629
C	21.28822	7.55687	2.57191
C	22.09939	8.73511	2.34742
O	19.14747	6.58811	2.77095
H	23.74065	6.88773	2.66708
H	26.95160	3.64009	3.21484
H	25.71928	5.74115	2.82398
H	22.01009	3.65359	3.35105
H	21.18355	5.48187	2.95679
C	0.04363	15.87067	3.39866
C	-0.82902	14.80996	3.20089
C	-0.34174	13.52688	3.02797
C	1.03246	13.29143	3.05180
C	1.91247	14.35353	3.25108
C	1.41123	15.63239	3.42207
N	1.46192	11.97301	2.87193
C	2.70732	11.52291	2.83768
C	3.07493	10.20092	2.65645
C	2.08920	9.15531	2.48019
O	5.34182	10.83295	2.80681
H	0.75538	11.24494	2.74837
H	-1.01779	12.69466	2.87235
H	2.98116	14.18948	3.27422
H	2.10149	16.45222	3.57633
H	3.50218	12.24865	2.96017
C	-0.88193	2.37933	1.39553
C	0.48865	2.16471	1.36233
C	1.36591	3.21891	1.54322
C	0.87717	4.50677	1.76077
C	-0.49874	4.72642	1.79675
C	-1.36508	3.66256	1.61334
N	1.81513	5.52899	1.93622
C	1.58419	6.82018	2.12301
C	2.55698	7.78994	2.29141
C	3.96579	7.46304	2.27749
O	0.86382	9.41825	2.48944
H	2.80732	5.28467	1.92048
H	2.43699	3.05690	1.51728
H	-0.89682	5.71716	1.96830
H	-2.43249	3.84183	1.64282
H	0.55064	7.14407	2.14429
C	23.98050	15.84298	0.99744

C	22.59505	15.86256	1.07404
C	21.89144	14.69437	1.30601
C	22.57163	13.48768	1.46447
C	23.96270	13.46287	1.38593
C	24.65440	14.63928	1.15440
N	21.80353	12.34220	1.69475
C	22.23372	11.10816	1.91167
C	21.42496	10.00536	2.12426
C	19.98252	10.11167	2.12301
O	23.35077	8.66340	2.34530
H	20.78577	12.43383	1.70162
H	22.05530	16.79321	0.95291
H	20.80989	14.70286	1.36812
H	24.50650	12.53533	1.50045
H	23.30532	10.94997	1.92505
H	25.71343	1.54035	3.67234
H	23.23899	1.57478	3.73477
H	-0.33747	16.87448	3.53368
H	-1.89803	14.98007	3.18032
H	-1.56841	1.55483	1.25402
H	0.88091	1.16969	1.19436
H	24.53110	16.75672	0.81572
H	25.73515	14.61171	1.09417

Supplementary Table 7: Atomistic coordinates of Re-TpBpy -n_propanol.

Space group: *P3;*

a = *b* = 29.712 Å, *c* = 9.4234 Å;

$\alpha = \beta = 90^{\circ}$, $\gamma = 120^{\circ}$.

Atom	*x (Å)*	*y (Å)*	*z (Å)*
Re	11.90630	11.62434	2.51252
C	13.34728	12.87699	2.65634
C	10.70622	13.11024	2.63530
Cl	11.85313	11.15375	5.00108
C	11.93848	11.86514	0.62711
O	14.22872	13.62434	2.73320
O	11.95970	12.03195	-0.52248
C	12.36680	8.57097	2.46530
C	13.03362	7.34795	2.47652
C	14.40565	7.31227	2.47218
C	15.12441	8.50731	2.45467
C	14.40366	9.69781	2.44060
N	13.07256	9.72446	2.44783
N	16.50632	8.45742	2.44959
C	17.36165	9.48123	2.46355
C	18.73202	9.35907	2.44970
C	19.38389	8.06148	2.41564
O	18.91614	11.71015	2.50207
H	16.96229	7.54094	2.43391
C	10.91775	8.69992	2.46339
N	10.42593	9.96032	2.43986
C	9.11168	10.15747	2.43069
C	8.19155	9.11228	2.44498
C	8.68571	7.80901	2.47030
C	10.04599	7.61402	2.47873
N	6.84848	9.43816	2.43322
C	5.81511	8.59586	2.46421
C	4.49171	8.97248	2.45092
C	4.09511	10.36854	2.39502
O	3.87220	6.69893	2.54400
H	6.57596	10.42454	2.39973
H	12.47678	6.42289	2.48958
H	14.93470	6.36784	2.48236
H	14.89880	10.65805	2.42199
H	16.94924	10.48225	2.48916
H	8.76698	11.18404	2.41323
H	8.02260	6.95584	2.48210

H	10.43444	6.60660	2.49837
H	6.03485	7.53606	2.50520
C	24.76904	2.88971	2.22634
C	25.45391	4.09655	2.22124
C	24.76135	5.29355	2.25852
C	23.36784	5.29346	2.30188
C	22.67577	4.08401	2.30785
C	23.38128	2.89383	2.26999
N	22.72415	6.53444	2.33863
C	21.42157	6.76927	2.36306
C	20.83528	8.02333	2.39733
C	21.63021	9.23375	2.41268
O	18.70857	7.00644	2.40184
H	23.29750	7.38084	2.34571
H	26.53596	4.10934	2.18779
H	25.29117	6.23856	2.25418
H	21.59506	4.06587	2.34335
H	20.76148	5.91038	2.35454
C	-0.27387	16.43984	2.20549
C	-1.15916	15.37166	2.23481
C	-0.68537	14.07275	2.27584
C	0.68756	13.82876	2.28802
C	1.58038	14.89849	2.25971
C	1.09234	16.19315	2.21842
N	1.09932	12.49278	2.32993
C	2.33579	12.02015	2.34076
C	2.67653	10.67841	2.38929
C	1.66925	9.63901	2.43575
O	4.95686	11.27640	2.35245
H	0.38072	11.76636	2.35888
H	-1.37126	13.23425	2.29864
H	2.64848	14.73007	2.27035
H	1.79204	17.01917	2.19666
H	3.14639	12.73816	2.31010
C	-1.45038	2.85445	2.82253
C	-0.08566	2.60513	2.79391
C	0.81477	3.65284	2.72234
C	0.35515	4.96856	2.67863
C	-1.01454	5.22371	2.70831
C	-1.90435	4.16581	2.77945
N	1.31579	5.98285	2.60783
C	1.11268	7.29001	2.54923
C	2.10711	8.25181	2.49334
C	3.50695	7.89552	2.49855

O	0.44952	9.92515	2.42745
H	2.30196	5.71531	2.60432
H	1.88145	3.46366	2.69980
H	-1.38992	6.23739	2.67764
H	-2.96700	4.37220	2.80223
H	0.08613	7.63581	2.54630
C	23.44139	16.48300	2.58323
C	22.05455	16.47459	2.62818
C	21.36202	15.27737	2.60302
C	22.05485	14.06960	2.53203
C	23.44789	14.07316	2.48661
C	24.12811	15.27837	2.51294
N	21.29573	12.89447	2.50883
C	21.73675	11.64677	2.46992
C	20.93991	10.51475	2.45098
C	19.49817	10.60264	2.47005
O	22.88169	9.17994	2.39436
H	20.27761	12.97604	2.52581
H	21.50479	17.40559	2.68345
H	20.27934	15.26388	2.63921
H	24.00313	13.14681	2.42971
H	22.80950	11.49906	2.45347
H	25.31104	1.95352	2.19736
H	22.83649	1.95816	2.27540
H	-0.64407	17.45620	2.17330
H	-2.22727	15.54817	2.22568
H	-2.15491	2.03494	2.87864
H	0.28373	1.58804	2.82746
H	23.98364	17.41931	2.60267
H	25.21026	15.27308	2.47690
O	9.96839	14.00103	2.69623

Supplementary Table 8: Molecular orbital transitions and their contributions to each excited state in TpBpy, including excitation energy (in eV), wavelength (in nm), oscillator strength (f) and orbital contributions (in %).

1 2.3455 eV 528.60 nm f= 1.66360 Spin multiplicity= 1:
H -> L 97.9%

2 2.4172 eV 512.92 nm f= 0.02990 Spin multiplicity= 1:
H-2 -> L 68.3%, H-1 -> L 22.8%, H-2 -> L+1 6.5%

3 2.4260 eV 511.06 nm f= 0.03840 Spin multiplicity= 1:
H-1 -> L 67.4%, H-2 -> L 22.5%, H-1 -> L+1 7.3%

4 2.5128 eV 493.41 nm f= 0.00000 Spin multiplicity= 1:
H -> L+1 70.7%, H-3 -> L 29.0%

5 2.7021 eV 458.84 nm f= 0.16450 Spin multiplicity= 1:
H-1 -> L+1 61.5%, H-2 -> L+1 21.2%, H-1 -> L+2 6.3%, H-1 -> L 5.6%

6 2.7238 eV 455.19 nm f= 0.21360 Spin multiplicity= 1:
H-2 -> L+1 61.4%, H-1 -> L+1 21.0%, H-2 -> L+2 6.6%, H-2 -> L 5.1%

7 2.7423 eV 452.12 nm f= 0.00720 Spin multiplicity= 1:
H-3 -> L 63.7%, H -> L+1 24.3%

8 2.8424 eV 436.20 nm f= 0.78090 Spin multiplicity= 1:
H-3 -> L+1 50.4%, H -> L+2 46.2%

9 2.8714 eV 431.79 nm f= 0.00030 Spin multiplicity= 1:
H-4 -> L 99.8%

10 2.8780 eV 430.80 nm f= 0.00020 Spin multiplicity= 1:
H-5 -> L 100.0%

11 2.8904 eV 428.95 nm f= 0.02010 Spin multiplicity= 1:
H-6 -> L 36.7%, H-3 -> L+1 33.4%, H -> L+2 27.6%

12 2.9792 eV 416.17 nm f= 0.07450 Spin multiplicity= 1:
H-2 -> L+2 47.6%, H -> L+4 30.9%, H-1 -> L+2 16.3%

13 2.9877 eV 414.98 nm f= 0.06480 Spin multiplicity= 1:
H -> L+3 45.0%, H-1 -> L+2 38.2%, H-2 -> L+2 12.9%

14 3.0132 eV 411.47 nm f= 0.27840 Spin multiplicity= 1:
H-6 -> L 50.5%, H -> L+2 17.6%, H-3 -> L+1 8.4%, H-2 -> L+4 8.2%, H-1 -> L+3 8.1%

15 3.0648 eV 404.54 nm f= 0.16330 Spin multiplicity= 1:
H -> L+4 58.7%, H-2 -> L+2 13.8%, H-2 -> L+5 13.0%

16   3.0873 eV   401.59 nm   f= 0.25710   Spin multiplicity= 1:
H -> L+3 43.9%, H-1 -> L+2 20.5%, H-1 -> L+5 15.8%, H-2 -> L+2 7.1%

17   3.0939 eV   400.74 nm   f= 0.00000   Spin multiplicity= 1:
H-4 -> L+1 99.7%

18   3.0990 eV   400.08 nm   f= 0.00380   Spin multiplicity= 1:
H -> L+5 59.0%, H-7 -> L 20.6%, H-3 -> L+2 12.6%

19   3.1004 eV   399.90 nm   f= 0.00000   Spin multiplicity= 1:
H-5 -> L+1 99.5%

20   3.1374 eV   395.18 nm   f= 0.00000   Spin multiplicity= 1:
H-2 -> L+3 75.5%, H-1 -> L+3 24.4%

Supplementary Table 9: Molecular orbital transitions and their contributions to each excited state in Re-TpBpy, including excitation energy (in eV), wavelength (in nm), oscillator strength (f) and orbital contributions (in %).

1 2.1372 eV 580.12 nm f= 0.02050 Spin multiplicity= 1:
H-1 -> L 95.2%

2 2.1480 eV 577.21 nm f= 0.03070 Spin multiplicity= 1:
H -> L 88.0%, H-2 -> L 7.7%

3 2.2232 eV 557.68 nm f= 1.60280 Spin multiplicity= 1:
H-2 -> L 87.5%, H -> L 8.4%

4 2.3480 eV 528.04 nm f= 0.14830 Spin multiplicity= 1:
H-3 -> L 95.9%

5 2.4190 eV 512.54 nm f= 0.01670 Spin multiplicity= 1:
H-4 -> L 81.5%, H-2 -> L+1 7.6%, H-5 -> L 6.1%

6 2.4827 eV 499.39 nm f= 0.02360 Spin multiplicity= 1:
H-5 -> L 90.9%

7 2.5812 eV 480.34 nm f= 0.03390 Spin multiplicity= 1:
H -> L+1 80.3%, H -> L+2 8.4%, H-2 -> L+1 7.4%

8 2.6335 eV 470.80 nm f= 0.00020 Spin multiplicity= 1:
H-6 -> L 99.8%

9 2.6347 eV 470.58 nm f= 0.00000 Spin multiplicity= 1:
H-7 -> L 99.8%

10 2.6563 eV 466.76 nm f= 0.12160 Spin multiplicity= 1:
H-1 -> L+1 74.8%, H-1 -> L+2 17.4%

11 2.6722 eV 463.98 nm f= 0.00080 Spin multiplicity= 1:
H-2 -> L+1 67.4%, H-4 -> L 7.6%, H -> L+1 6.9%

12 2.7690 eV 447.76 nm f= 0.11400 Spin multiplicity= 1:
H-8 -> L 44.0%, H-2 -> L+2 28.0%, H-3 -> L+1 19.4%

13 2.7819 eV 445.68 nm f= 0.12630 Spin multiplicity= 1:
H-3 -> L+1 67.6%, H-1 -> L+2 13.5%, H-8 -> L 10.2%

14 2.7834 eV 445.44 nm f= 0.11580 Spin multiplicity= 1:
H-1 -> L+2 62.2%, H-1 -> L+1 11.5%, H-3 -> L+1 10.2%, H-2 -> L+2 5.0%

15 2.8025 eV 442.41 nm f= 0.00130 Spin multiplicity= 1:
H-9 -> L 99.7%

16 2.8132 eV 440.72 nm f= 0.44470 Spin multiplicity= 1:
H-4 -> L+1 41.1%, H-2 -> L+2 25.6%, H-8 -> L 23.4%

17 2.8790 eV 430.65 nm f= 0.00650 Spin multiplicity= 1:
H-5 -> L+1 96.4%

18 2.8979 eV 427.84 nm f= 0.36030 Spin multiplicity= 1:
H -> L+2 70.4%, H-2 -> L+2 7.6%, H -> L+1 6.2%

19 2.9265 eV 423.66 nm f= 0.02490 Spin multiplicity= 1:
H-4 -> L+1 41.7%, H-2 -> L+2 19.0%, H-3 -> L+2 13.7%, H-8 -> L 7.3%

20 2.9936 eV 414.16 nm f= 0.00540 Spin multiplicity= 1:
H-3 -> L+2 77.3%, H-4 -> L+2 10.1%

Supplementary References

1. Chandra, S. *et al.* Chemically stable multilayered covalent organic nanosheets from covalent organic frameworks via mechanical delamination. *J. Am. Chem. Soc.* **135**, 17853–17861 (2013).

2. Zhong, W. *et al.* A Covalent Organic Framework Bearing Single Ni Sites as a Synergistic Photocatalyst for Selective Photoreduction of CO2 to CO. *J. Am. Chem. Soc.* **141**, 7615–7621 (2019).

3. Aiyappa, H. B., Thote, J., Shinde, D. B., Banerjee, R. & Kurungot, S. Cobalt-Modified Covalent Organic Framework as a Robust Water Oxidation Electrocatalyst. *Chem. Mater.* **28**, 4375–4379 (2016).

4. Chandra, S. *et al.* Interplaying Intrinsic and Extrinsic Proton Conductivities in Covalent Organic Frameworks. *Chem. Mater.* **28**, 1489–1494 (2016).

5. Li, S.-Y. *et al.* Rhenium-functionalized covalent organic framework photocatalyst for efficient CO2 reduction under visible light. *Microporous Mesoporous Mater.* **285**, 195–201 (2019).
